# Sugar and iron: Toward understanding the antibacterial effect of ciclopirox in *Escherichia coli*

**DOI:** 10.1371/journal.pone.0210547

**Published:** 2019-01-11

**Authors:** Zachary C. Conley, Kimberly M. Carlson-Banning, Ashley G. Carter, Alejandro de la Cova, Yongcheng Song, Lynn Zechiedrich

**Affiliations:** 1 Verna and Marrs McLean Department of Biochemistry and Molecular Biology, Baylor College of Medicine, Houston, Texas, United States of America; 2 Department of Virology and Microbiology, Baylor College of Medicine, Houston, Texas, United States of America; 3 Center for Environmental and Human Toxicology, University of Florida College of Veterinary Medicine, University of Florida, Gainesville, Florida, United States of America; 4 Department of Pharmacology and Chemical Biology, Baylor College of Medicine, Houston, Texas, United States of America; Universidade Nova de Lisboa, PORTUGAL

## Abstract

New antibiotics are needed against antibiotic-resistant gram-negative bacteria. The repurposed antifungal drug, ciclopirox, equally blocks antibiotic-susceptible or multidrug-resistant *Acinetobacter baumannii*, *Escherichia coli*, and *Klebsiella pneumoniae* clinical isolates, indicating that it is not affected by existing resistance mechanisms. Toward understanding how ciclopirox blocks growth, we screened *E*. *coli* mutant strains and found that disruption of genes encoding products involved in galactose salvage, enterobacterial common antigen synthesis, and transport of the iron binding siderophore, enterobactin, lowered the minimum inhibitory concentration of ciclopirox needed to block growth of the mutant compared to the isogenic parent strain. We found that ciclopirox induced enterobactin production and that this effect is strongly affected by the deletion of the galactose salvage genes encoding UDP-galactose 4-epimerase, *galE*, or galactose-1-phosphate uridylyltransferase, *galT*. As disruption of ECA synthesis activates the regulation of capsular synthesis (Rcs) phosphorelay, which inhibits bacterial swarming and promotes biofilm development, we test whether ciclopirox prevents activation of the Rcs pathway. Sub-inhibitory concentrations of ciclopirox increased swarming of the *E*. *coli* laboratory K12 strain BW25113 but had widely varying effects on swarming or surface motility of clinical isolate *E*. *coli*, *A*. *baumannii*, and *K*. *pneumoniae*. There was no effect of ciclopirox on biofilm production, suggesting it does not target Rcs. Altogether, our data suggest ciclopirox-mediated alteration of lipopolysaccharides stimulates enterobactin production and affects bacterial swarming.

## Introduction

It is estimated that 23,000 people a year die from infections of antibiotic-resistant bacteria [1]. According to the Centers for Disease Control and Prevention, *Enterobacteriaceae* are among the three most urgent of these antibiotic-resistant threats [1]. Among many approaches for fighting the “super-wicked” problem of antibiotic resistance [2], one is the development of novel antibacterial agents, especially drugs with new mechanisms of action, particularly against these “urgent threat” pathogens [1,3].

Novel antibacterial therapies can be found by repurposing off-patent drugs already approved by the Food and Drug Administration for other treatments. This repurposing allows faster movement through the drug development pipeline. One promising repurposed drug is ciclopirox, an off-patent antifungal agent with a good safety profile and a long and successful history of antifungal use [4]. Despite being used for over 30 years, no fungal resistance has been identified [4,5]. Additionally, sub-inhibitory concentrations of ciclopirox failed to induce resistance in *Candida albicans*, even after six months of culturing (discussed in [4]).

Despite only being marketed for topical use, in part because of the large quantity of antibiotics on the market at the time of its development, ciclopirox has been assessed for other purposes, including systemically [6–10]. Acute toxicity studies performed in mice and rats revealed LD_50s_ ranging from 1,700 to > 2,500 mg/kg (oral and subcutaneous), 71–79 mg/kg (intravenous), and 83–172 mg/kg (intraperitoneal). Chronic and subacute toxicity studies showed that oral administration of 10 mg/kg/day for three months was well tolerated in rats and dogs, although long-term oral doses of ≥30 mg/kg/day resulted in toxicity in dogs. In a phase I study to treat patients with hematologic malignances, daily doses of 40 mg/m^2^ ciclopirox olamine were well tolerated, although gastrointestinal toxicities occurred in patients receiving 80 mg/m^2^ four times a day [11]. These favorable outcomes have led to ciclopirox currently being investigated as a treatment for multiple types of cancer [11–19], diabetes mellitus [20–22], herpes simplex virus [23], congenital erythropoietic porphyria [24] and human immunodeficiency virus (HIV) infection [25,26].

The excellent safety profile and lack of fungal resistance also make ciclopirox a prime candidate for repurposing as an antibacterial agent [27–29]. Ciclopirox blocks growth of *A*. *baumannii*, *E*. *coli*, and *K*. *pneumoniae* clinical isolates independently of antibiotic resistance status [27]. In particular, thirty non-clonal *E*. *coli* isolates with a range of antibiotic resistance phenotypes (from pan-antibiotic susceptible to resistant to 1–8 classes of antibiotics), with ciprofloxacin MICs ranging from 0.008 to 500 μg/mL, were all inhibited by 5–15 μg/mL of ciclopirox [27]. This finding suggests that ciclopirox has a novel drug target in these gram-negative bacteria for which bacteria have not yet developed resistance.

Ciclopirox has iron chelation properties, but the mechanism of inhibition differs between humans and fungi. In yeast, ciclopirox iron chelation leads to increased sensitivity to oxidative stress, reduced expression and activity of catalase, suspected loss of activity in iron-dependent enzymes, and cell death through production of reactive oxygen species [30–32]. In *C*. *albicans*, however, 97% of ciclopirox is found bound to cell structures and organelles and the drug modifies particle distribution within the plasma membrane of both *C*. *albicans and* dermatophytes (discussed in [32]). As an HIV infection treatment, ciclopirox binds iron in the active site of deoxyhypusine hydroxylase to prevent post-translational modification of lysine residues into the rare amino acid hypusine [26]. Hypusine is only found in eukaryotic translation initiation factor 5A (eIF5a), and is essential in some higher eukaryotes [26,33,34]. Without mature eIF5a, HIV-infected cells are less able to suppress intrinsic apoptotic pathways, causing the infected cells to undergo apoptosis [26]. As an anticancer treatment, ciclopirox iron sequestration leads to inhibition of Wnt signaling and cell cycle progression, the latter due to sequestering iron from ribonucleotide reductase and deoxyhypusine hydroxylase [11].

Some information is known about ciclopirox as an antibacterial agent. Unlike yeast [30–32], bacteria grown in ciclopirox do not become more susceptible to hydrogen peroxide-induced oxidative stress [27]. Excessive iron in the growth medium (4–16 times the concentration found in human serum) rescues *E*. *coli* growth in otherwise inhibitory concentrations of ciclopirox [27]. *E*. *coli* grown in sub-inhibitory concentrations of ciclopirox have decreased levels of both the highly variable oligosaccharide O-antigen component and the more conserved polysaccharide core component of lipopolysaccharide (LPS) [27]. Furthermore, from a screen of 4,267 gene overexpression plasmids in the *E*. *coli* ASKA library, the overexpression of *galE* rescues growth of *E*. *coli* in ciclopirox [27]. UDP-galactose 4-epimerase, GalE, functions in the galactose salvage pathway to convert UDP-glucose into UDP-galactose. Without GalE, exogenous galactose is required for completion of the outer core oligosaccharide and production of O-antigen side chains [35]. Ciclopirox, however, does not directly inhibit purified GalE activity [27].

Continuing to expand our understanding of the phenotypic changes resulting from ciclopirox treatment could potentially lead to identification of the ciclopirox mechanism of action in bacteria. In turn, this mechanism could be exploited as a new drug target, or lead to identification of other targets in the same pathway. Here we screened gene deletion strains from the *E*. *coli* Keio collection for decreased ciclopirox minimum inhibitory concentrations (MICs) required to block growth and identified two pathways affected by the drug. One involves utilization (synthesis and transport) of the siderophore, enterobactin, and the other involves synthesis of the polysaccharide, enterobacterial common antigen.

## Materials and methods

### Chemicals and reagents

Calcium chloride, hydrochloric acid, anhydrous dextrose (D-glucose), dimethyl sulfoxide (DMSO), light mineral oil, sodium hydroxide, sodium chloride, agar, and sodium phosphate dibasic were purchased from Fisher Scientific (Waltham, USA). Sodium molybdate, sodium nitrite, and ciclopirox were from Santa Cruz Biotechnologies (Dallas, USA). Ammonium chloride, ampicillin, chloramphenicol, ciprofloxacin, dimethylformamide, iron (III) chloride, 1,10-phenanthroline, sodium dodecyl sulfate (SDS), and 3-(4,5-dimethylthiazol-2-yl)-2,5-diphenyl tetrazolium bromide (MTT) were from Sigma Aldrich (St. Louis, USA). Mueller Hinton (MH) broth, tryptic soy broth (TSB), tryptone, and casamino acids were from DIFCO (Sparks, USA). Potassium phosphate monobasic was from Macron Fine Chemicals (Center Valley, USA). Ethanol was purchased from Decon Labs, Inc. (King of Prussia, USA). A QIAprepSpin MiniPrep Kit was purchased from QIAGEN (Valencia, CA). Yeast extract and tris base was purchased from Millipore Sigma (Burlington, USA). Eiken agar was purchased from Eiken Chemical Co. (Tokyo, Japan). Kern River crude (13 Deg API) oil was provided by Donald S. Mims (Chevron, Houston, TX USA).

Ampicillin, ciclopirox, ciprofloxacin, chloramphenicol and 1,10-phenanthroline stocks were stored at -20° C. Ampicillin and ciprofloxacin were stored in H_2_O at stock concentration of 50 mg/mL. Ciclopirox was stored in DMSO at a concentration of 100 mM. Chloramphenicol was stored in 100% ethanol at a concentration of 20 mg/mL. 1,10-phenanthroline was stored in 100% ethanol at a concentration of 100 mM.

### Bacterial strains and plasmids

The *E*. *coli* Keio knock-out strains, BW25113 (the isogenic parent strain), and *E*. *coli* strain AG1 containing ASKA plasmids were obtained from the National BioResource Project Center [36,37]. *A*. *baumannii* strain ATTC17978, *E*. *coli* strain ATTC BAA-1743 SMS-3-5, *K*. *pneumoniae* strain ATTC 11296, and *Pseudomonas aeruginosa* strain ATTC 27853 were obtained from the American Type Culture Collection (Manassas, USA). Clinical isolate *E*. *coli* ELZ4486 was taken from a collection of clinical isolates previously described [38–40]. *E*. *coli s*trains CAG 18556, CAG 18565, CAG 18619, and the isogenic parent MG1655 were generously provided by Dr. Carol A. Gross.

### Bacterial growth media

Four different growth media were used for these experiments. For measuring antibiotic susceptibility and siderophore production, bacteria were grown in MH. To measure siderophore production under low iron conditions, cells were grown in M9 minimal medium (M9). For the biofilm formation assay, cells were grown in tryptic soy broth (TSB). All other experiments were performed using lysogeny broth (LB).

### Antibiotic susceptibility determinations

Minimum inhibitory concentrations (MICs) were measured using a broth dilution protocol performed in accordance with Clinical Laboratory Standards Institute (CLSI) [41]. The protocol was modified to include a wider range of drug concentrations (0.125–6 μg/mL for ampicillin, 5–20 μg/mL for ciclopirox, 0.005–0.05 μg/mL for ciprofloxacin, and 5–15 μg/mL for 1,10-phenanthroline). Each MIC measurement was done at least in triplicate. In addition to reporting the MIC ranges, we also, when relevant, report averaged MIC values and assessed statistical relevance using the Student’s t-test.

### Keio knock-out strain complementation with ASKA plasmid

Overnight cultures of Keio strains Δ*tonB* and Δ*fepA* as well as *E*. *coli* strain AG1 containing either the empty ASKA plasmid (pCA24N) or ASKA plasmids encoding the *tonB* or *fepA* genes were diluted 1:100 into fresh LB medium and grown shaking at 37° C for 2 hours. 1.5 mL of culture was transferred to Eppendorf tubes and centrifuged for 1 minute to pellet cells. Supernatant was removed via aspiration and cells were resuspended in 100 μL of cold 100 mM CaCl_2_, 10 mM Tris-Cl, pH 7.5. 25 ng of each ASKA plasmid, which were purified using the QIAprep Spin MiniPrep Kit, were added to each tube. Each Keio strain received the empty ASKA plasmid or the plasmid containing the complementing gene. Cells were incubated for 1 hour on ice, placed for 5 minutes in a water bath at 37° C, and returned to ice. 1 mL of LB medium was added to each tube and the tubes were incubated for 1 hour at 37° C. Cells were centrifuged for 1 minute and the supernatant was removed. Pellets were resuspended in 100 μL of LB medium and then spread onto LB agar plates containing 20 μg/mL chloramphenicol to select for colonies containing the ASKA plasmid. Plates were incubated overnight at 37° C.

### Siderophore quantification

Overnight bacteria cultures were diluted 1:100 in MH medium and grown, with shaking, at 37° C to an OD_600_ = 0.1. Cultures were then diluted 1:100 into fresh medium, and grown, shaking, at 37° C for 17 hours. Cell densities were measured at OD_600_. One mL of culture was centrifuged and the supernatant was used for the chemical assays. To test the effect of drugs on siderophore production, 5 μg/mL of ciclopirox or 1.5 μg/mL of 1,10-phenanthroline was added to MH during the 17 hours of shaking growth. In low iron conditions M9 was used instead of MH.

Catechol siderophores were quantified using a slightly modified Arnow assay [42]. 1 mL of overnight cell culture was centrifuged. 400 μL of supernatant was combined with equal parts 0.5 M HCl, nitrite-molybdate reagent (10% sodium nitrite, 10% sodium molybdate), and 1 M NaOH. The mixture was shaken after each reagent was added. The resulting mixture was stable for 1 hour, with an absorption maximum of 510 nm. The absorbance at 510 nm was divided by the OD_600_ of the culture and the ratio was used as a measurement of the amount of catechol siderophore per cell density.

### Bacterial swarming

Motility was measured under conditions that are inducive to bacterial swarming. Thus the surface motility for *P*. *aeruginosa* and *E*. *coli* is considered bacterial swarming while the movement of non-flagellated strains is referred to as surface motility. Swarming and surface motility was assessed on LB growth medium with 0.5% glucose and 0.5% Eiken agar. For the clinical isolate *E*. *coli*, *A*. *baumannii*, and *K*. *pneumoniae*, some of which were robust swarmers, regular 0.5% bacto agar was used. For some experiments, as indicated, various concentrations of ciclopirox (μg/mL), 1,10-phenanthroline (μg/mL), or ferric chloride (μM) were added to the agar following sterilization. 25 mL liquid agar were poured per plate. The agar was allowed to solidify with the lid slightly opened for 2 hours. Overnight bacteria cultures were diluted 1:100 in LB growth medium and grown, with shaking at 37° C, to an OD_600_ ≈ 0.6. Eight μL of culture were inoculated on the agar surface at the center of each plate without piercing. Plates were incubated overnight for ~18 hours, uninverted.

Swarmed bacteria were photographed and analyzed using the Image J software. Images were converted to black and white. The threshold of the image was adjusted to only read the visual cell growth as black pixilation, as given as a percentage of the total area of the plate measured in centimeters. The swarmed area was calculated by multiplying the total area in centimeters by the percentage of the plate with visual cell growth.

### Biofilm forming assay

Overnight bacterial cultures were diluted 1:100 in TSB and placed in a 96-well plate. Increasing concentrations of ciclopirox, diluted into TSB for a final volume of 100 μL, were added per well. Plates were incubated for 24 hours at 37° C. Medium was then removed and plates washed three times with 150 μL per well of PBS. 100 μL of PBS and 10 μL of 5 mg/mL MTT were added to each well and incubated for 4 hours at 37° C. 100 μL of extraction buffer (20% SDS [wt./vol.], 50% dimethylformamide in water, pH = 4.7) were added to each well and the plates were incubated 18 hours at 37° C. Absorbance values at 560 nm indicated viable biofilm forming cells.

### Drop collapse assay

Overnight bacterial cultures were diluted 1:100 in MH growth medium and grown, with shaking, at 37° C to an OD_600_ = 0.1. Cultures were then diluted 1:100 into fresh MH medium with increasing concentrations of ciclopirox (μg/mL), and grown, shaking, at 37° C for 17 hours. Cell densities were measured at OD_600_. One mL of culture was centrifuged and the supernatant was separated from the cell pellet. An adapted drop collapse assay was used [43]. Two μL of mineral oil were spotted on the underside of a 96-well microtiter plate lid and allowed to equilibrate for 1 hour at room temperature. 5 μL of supernatant were added to the thin layer of mineral oil formed at each spot. The shape of the drops was inspected after 1 minute. Biosurfactants result in flatter drops.

### Oil displacement assay

Overnight bacteria cultures were diluted 1:100 in MH growth medium and grown, with shaking, at 37° C to an OD_600_ = 0.1. Cultures were then diluted 1:100 into fresh MH medium with increasing concentrations of ciclopirox (μg/mL), and grown, shaking, at 37° C for 17 hours. Cell densities were measured at OD_600_. One mL of culture was centrifuged and the supernatant was separated from the cell pellet. An adapted oil displacement assay was used [44,45]. A 150 mm petri dish was placed over a 1 cm^2^ grid drawn on paper. The dish was filled with 42 mL of distilled water, and swirled slightly to ensure coverage of the entire surface. Using a Pasteur pipette, a drop of viscous crude oil (Kern River crude (13 Deg API)) was placed on the surface of the water, allowing a thin membrane of oil to form. Ten μL of supernatant was dotted onto the thin oil surface and the displaced oil was measured. Triton X-100 served as a positive control.

### Statistical analyses

A Student’s T test was used to assess significance for MIC measurements, siderophore production, swarming, and biofilm production. For MICs and siderophore quantification, results were considered significant when *p* < 0.005 and, because of the vastly increased variability of the assay, results for swarming and biofilm production were considered significant with *p* < 0.05.

## Results

### Effect of gene deletions on ciclopirox MICs

In our previous work we found that overexpression of *galE* rescues *E*. *coli* growth in the presence of otherwise inhibitory concentrations of ciclopirox and that the deletion of any one of a number of additional genes in the galactose salvage pathway lowers ciclopirox MICs [27]. Because GalE is not the direct ciclopirox target [27], we inferred that ciclopirox affects LPS [27] to render bacteria more vulnerable to the availability of sugar monomers provided by the galactose salvage pathway. Because ciclopirox binds iron, it was important to distinguish whether the mutant strains were affected by ciclopirox binding iron or through a different mechanism.

We selected a group of 96 mutant strains from the Keio collection of 3,985 deletions of non-essential genes in K-12 *E*. *coli* [36,46]. The deleted genes were involved in sugar metabolism, LPS synthesis, iron salvage, two-component regulation, and synthesis of the translation factor elongation factor P (S1 Table).

Forty-three of these mutant strains had significantly lower (*p* < 0.005) ciclopirox MICs than the isogenic parent strain (S1 Table). In the Keio mutant collection the replacement of each gene is with the kanamycin resistance gene [46]. By measuring ciclopirox MICs in strains CAG18556, CAG18565, and CAG18619, containing the kanamycin resistance gene, compared to the isogenic parent MG1655, we demonstrated that this gene itself had no effect on the ciclopirox MIC (data not shown). In addition, the MIC was restored in the Δ*fepA* and Δ*tonB* Keio strains complemented with the ASKA plasmids containing *fepA* or *tonB* respectfully (data not shown). Therefore, the MIC changes we saw resulted from the deletion of the genes of interest.

To separate the effects of iron chelation by ciclopirox from additional antibiotic effects, we measured 1,10-phenanthroline MICs in the 43 mutants with lower ciclopirox MICs, as 1,10-phenanthroline chelates metal (including iron). Nineteen of these strains also had lower (*p* < 0.005) 1,10-phenanthroline MICs (S1 Table). To distinguish those gene deletions specifically affected by ciclopirox from those involved with resistance to antibiotics in general, we compared our gene list to previously published data of 283 Keio deletion strains more susceptible to at least one of 14 tested antibiotics [47]. Nine of our 43 strains appeared on this list (S1 Table).

To assess whether antibiotics in general affected any of the mutant strains on our list, we measured the ampicillin and ciprofloxacin MICs for 15 representative strains from each biochemical pathway (one each from each pathway with a change in the ciclopirox MIC but no change in the 1,10-phenanthroline MIC) and verified that these deletion mutants were not generally affected by antibiotics (S2 Table). These efforts resulted in 21 deletion mutants with lower ciclopirox MICs that could not be explained by response to iron chelation or antibiotics.

### Biochemical pathways affected by ciclopirox

Of the 21 deletion mutants specifically affected by ciclopirox, four (Δ*galK*, Δ*galM*, Δ*galP*, Δ*ugd*) are from the Leloir pathway for galactose salvage. This pathway allows *E*. *coli* to utilize external galactose for metabolism and as a subunit precursor for synthesis of glycolipids [48–50]. Two of the 21 deletion mutants (Δ*waaB* and Δ*waaO*) strains lack the genes that encode proteins involved in the synthesis of the structurally conserved sugar core region of LPS [51]. In particular, *waaB* encodes UDP-D-galactose (glucosyl) LPS-1, 6-D-galactosyltransferase, and *waaO* encodes UDP-D-glucose:(glucosyl) LPS α-1,3-glucosyltransferase. These two enzymes work together to add a galactose and glucose, respectively, to the first glucose residue in the outer core of LPS [51]. These findings agree with our previous data [27] that showed disrupting synthesis of core LPS or galactose salvage increases cell susceptibility to ciclopirox but extend the data to show, importantly, that these effects are independent of iron chelation.

BW25113, the isogenic parent strain for the Keio collection, does not express the highly variable O-antigen component of LPS as a consequence of disruption of the gene encoding rhamnosyltransferase needed for O-antigen unit assembly [52]. Thus, most BW25113 mutants tested that encode proteins involved in this aspect of LPS assembly had no change in the ciclopirox MICs compared to the parent strain, as expected. The strain with deleted *wzzB*, which encodes the protein that regulates the chain length of O-antigen [53], may have caused a slight MIC change. It is not yet clear how chain-length regulators interact with other O-antigen assembly proteins [54] and it is, thus, unclear why Δ*wzzB* had a slightly lower ciclopirox MIC than the isogenic parent strain.

**Fig 1:**
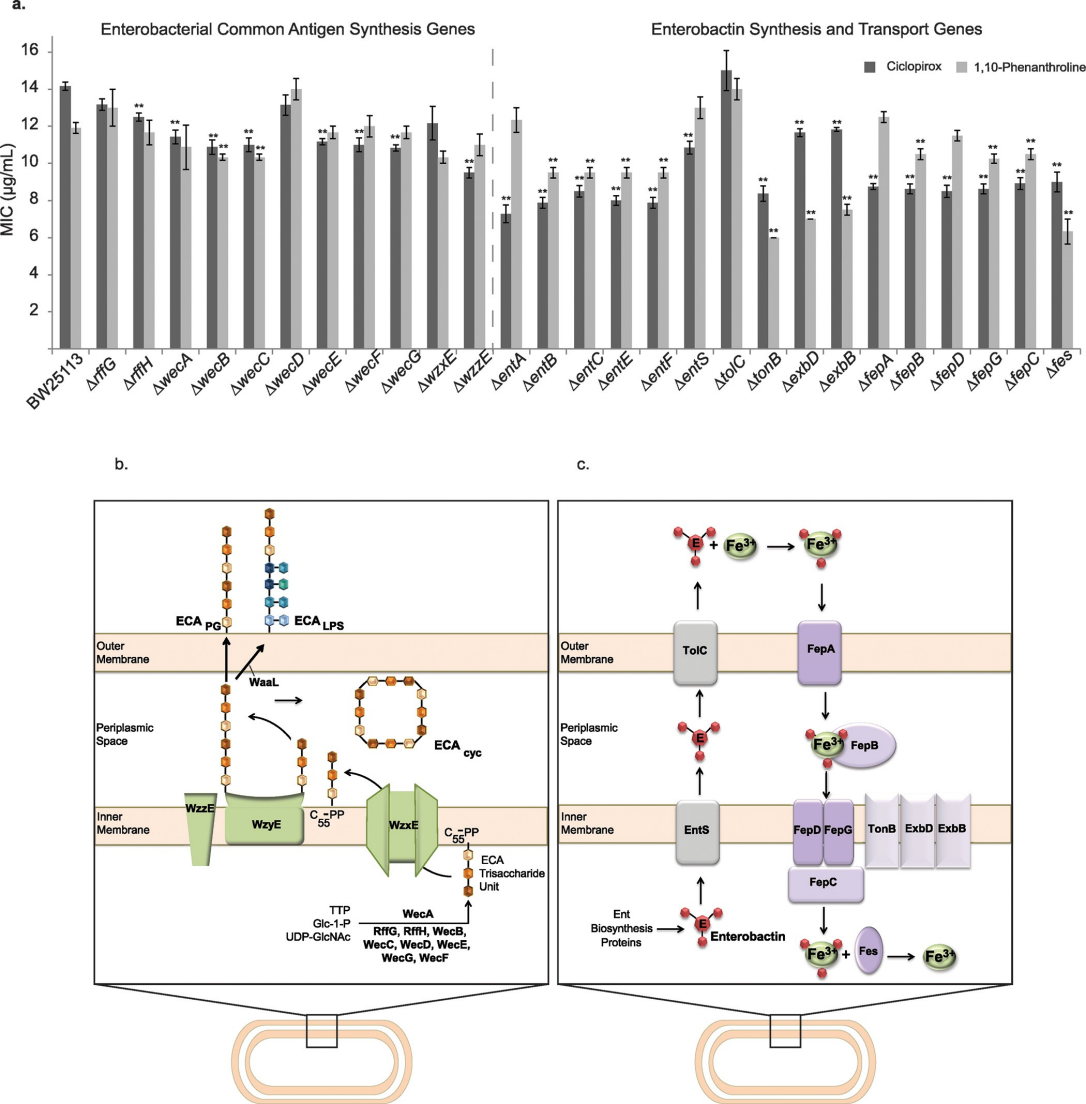
Deletion of ECA synthesis or enterobactin transport pathway genes lower ciclopirox MICs. (a) Ciclopirox and 1,10-phenanthroline MICs of the *E*. *coli* Keio parent strain BW25113 and the isogenic deletion mutant strains. Student’s t-test was used to assess significance of each mutant relative to BW25113 (** *p* < 0.005). (b) Schematic of the Enterobacterial Common Antigen synthesis pathway (adapted from a combination of [54–56]). The Wec and Rff family proteins assemble the ECA trisaccharide unit, which consists of repeating *N*-acetyl-D-glucosamine, *N*-acetyl-D-mannosaminuronic acid, and 4-acetamido-4,6-dideoxy-D-galactose on the C55 isoprenoid lipid carrier undecaprenol diphosphate (C_55_-PP). ECA polymers are assembled using a designated flippase (WzxE) and polymerase (WzyE), the length of which is controlled by a chain-length regulator protein (WzzE). ECA is covalently linked to the phosphoglyceride (ECA_PG_), in the periplasm in a cyclic form (ECA_Cyc_) or is attached to the core LPS by the RfaL protein (ECA_LPS_). (c) Schematic of the Enterobactin Transport Pathway (adapted from a combination of [57–60]). Enterobactin, synthesized by the Ent family proteins, is transported extracellularly by the transport proteins EntS and TolC. The channel protein TolC is complexed with RND transporters AcrAB, AcrAD or MdtABC (not shown). Ironbound enterobactin is then transported back into the bacterial cell through an outer membrane gated channel protein, FepA, a periplasmic protein, FepB, and an inner membrane ABC transporter, FepC-FepD-FepG. FepA is powered by an inner membrane protein complex, TonB-ExbB-ExbD. Once in the cytoplasm, iron is freed from the siderophore by the enterochelin esterase Fes.

Five strains (Δ*rffH*, Δ*wecG*, Δ*wecF*, Δ*wecA*, and Δ*wzzE*) are mutants of gene products involved in the synthesis and/or transport of ECA, a surface glycolipid of repeating trisaccharide units found in Enterobacteriaceae (Fig 1A and 1B). The *rff* gene family products are responsible for ECA trisaccharide subunit synthesis [55,56]. In particular, the gene *wecA* encodes UDP-*N*-acetylglucosamine—undecaprenyl-phosphate *N-*acetylglucosaminephosphotransferase, a protein that initiates biosynthesis of ECA subunits by transferring *N-*acetylglucosamine-1-phosphate onto the lipid carrier C55 isoprenoid lipid carrier undecaprenyl phosphate upon which the remaining subunit is assembled [54]. The gene *wzzE* encodes enterobacterial common antigen polysaccharide co-polymerase, the protein responsible for regulating the length of ECA polysaccharide chains [54]. The majority of gene deletion mutants from the ECA synthesis/transport pathway had lower ciclopirox MICs than the parent strain. Therefore, ciclopirox may affect the synthesis or transport of ECA.

Whereas the majority of ECA is found covalently linked directly to the phosphoglyceride or is found in the periplasm in a cyclic form, it also can be attached to the core LPS in strains that do not produce O-antigen, such as the BW25113 based Keio knock-out mutant strains (Fig 1B) [54]. Without *waaL*, the gene encoding O-antigen ligase, bacteria are unable to attach ECA subunits to core LPS [61,62]. The Δ*waaL* strain showed no significant change in ciclopirox MIC, suggesting that it is the loss of the ECA directly attached to the bacterial membrane (and potentially the cyclic ECA) that increases sensitivity of this mutant to ciclopirox.

Four mutant strains (Δ*fepA*, Δ*fepD*, Δ*entA*, and Δ*entS*) with decreased ciclopirox MICs are from pathways for synthesis or transport of enterobactin, a siderophore secreted by bacteria to salvage iron, yet these mutants had no effect on 1,10- phenanthroline MICs (Fig 1A and 1C). The *ent* gene family (*entA*, *entB*, *entC*, *entE*, and *entF*) encode the proteins responsible for synthesis of enterobactin (*entA* in particular encodes 2,3-dihydro-2,3-dihydroxybenzoate dehydrogenase) [57]. The protein encoded by *entS* is responsible for transporting synthesized enterobactin across the inner membrane en route to leaving the cell [58,59,63]. The *fep* gene family (*fepA*, *fepB*, *fepC*, *fepD*, and *fepG*) encodes the system that transports enterobactin bound to iron into the cell where the enterobactin is hydrolyzed by enterochelin esterase (encoded by *fes*) [57,60,64]. FepA, in particular, is the outer membrane gated channel protein, which transports enterobactin using the proton motive force facilitated by the TonB protein complex (encoded by *tonB*, *exbB*, and *exbD*). FepD is a subunit of the ABC transporter complex used to transport enterobactin across the inner membrane. All gene deletion strains in these pathways had lower ciclopirox MICs, signifying that cells unable to use enterobactin to scavenge iron are more sensitive to ciclopirox.

We expected to see lower ciclopirox MICs for deletions of enterobactin synthesis and transport genes compared to the parent strain because disruption of iron salvage pathways will make bacteria sensitive to lower iron concentrations and ciclopirox is an iron chelator that will lower free iron concentrations in the bacterial growth medium. Yet, neither the deletion of genes encoding proteins required for transport of the siderophores ferrichrome or citrate [60] nor the gene encoding the master ferric uptake regulator, *fur*, which controls siderophore synthesis [65], resulted in lower ciclopirox MICs (S1 Table). The Δ*fecE* mutant strain had a statistically relevant *higher* ciclopirox MIC. Thus, our further research focused on how ciclopirox interacts with transport of the siderophore enterobactin, and not transport of other siderophores, since the ciclopirox MICs only changed when enterobactin (and not other siderophore) transport genes were deleted. This result may occur because enterobactin is the primary siderophore utilized by *E*. *coli* [66].

In the Δ*fur* mutant strain, where there is increased production of enterobactin and its associated transport proteins, there was no change in ciclopirox MIC (S1 Table). This finding demonstrates that neither an increase in enterobactin concentration or enterobactin transport proteins alleviate the antimicrobial activity of ciclopirox. Therefore, ciclopirox may interfere with enterobactin transport into the bacteria across the membrane, such that bacteria lacking transport proteins are more sensitive to the drug but overexpression of the transport system and enterobactin is not enough to cause resistance.

The strain lacking *fetB* also had a lower ciclopirox MIC. The putative ABC-type transporter, FetAB, is an iron exporter, which reduces oxidative stress by mediating iron homeostasis. FetB encodes the inner membrane metal resistance protein [67]. If ciclopirox targets the exporter, we would expect either mutant to have higher ciclopirox MICs. Deleting *fetA*, however, caused no change in ciclopirox MIC, indicating, perhaps, that FetB has an additional (unknown) role in *E*. *coli*.

Two genes (*phoQ*, *soxS*) correspond to two-component transcriptional regulators, which respond to different environmental stresses at the bacterial cell surface (S1 Table). The PhoPQ system (encoded by *phoP* and *phoQ*) responds to low magnesium concentrations or antimicrobial peptides whereas the SoxRS system (*soxR*, *soxS*) responds to superoxide stress [63,68,69]. The Δ*phoP* strain had a lower ciclopirox MIC compared to the parent strain, but was excluded from the final set of deletion mutants as it was also more sensitive than the isogenic parent to six other antibiotics [47]. The PhoPQ system positively regulates LPS modification by controlling the expression of LPS modifying enzymes [68]. Meanwhile, as part of the superoxide response, the SoxRS system modifies iron metabolism by inducing expression of *fur* [63,69]. Changes to LPS and iron metabolism may contribute to the lower ciclopirox MICs of the Δ*phoQ* and Δ*soxS* strains.

Although the eukaryotic ciclopirox target deoxyhypusine hydroxylase does not have a bacterial homolog, the protein this hydroxylase modifies, eIF5α, has 64% percent homology with bacterial elongation factor P (EF-P) [34]. As such, we examined the possibility that ciclopirox targets the post-translational modification of EF-P the same way that it targets the modification of eIF5α in eukaryotes. Indeed, two strains (Δ*empA*, Δ*efp)* from this pathway were more susceptible than the isogenic parent strain to ciclopirox but not 1,10-phenanthroline (S1 Table). The gene *efp* encodes elongation factor P and *empA* encodes EF-P-lysine lysyltransferase, a protein involved in EF-P lysine residue modification [34]. If ciclopirox inhibits the post-translational modification of EF-P, it would be expected that ciclopirox would not affect the Δ*efp* strain, as a strain without EF-P would have nothing to inhibit. For the Δ*efp* strain there would be either no change in ciclopirox MIC or a higher MIC but not a lower MIC. Therefore, we must consider other possibilities for why three of the four genes in this pathway increased sensitivity to ciclopirox. These results are most likely a consequence of downstream functions of the deletion of EF-P. EF-P alleviates ribosomal stalling for some proteins with consecutive proline residues, while loss of EF-P affects protein expression and disrupts integrity of the bacterial membrane [34,70]. The lower ciclopirox MIC in the Δ*efp* mutant could result from this change in protein expression.

### Ciclopirox stimulates enterobactin production

Altering the composition of O-antigen leads to decreased siderophore intake in *Vibrio anguillarum* through a resulting decrease of transport protein in the outer membrane [71]. These findings combined with the fact that ciclopirox affects O-antigen and LPS synthesis [27], led us to hypothesize that ciclopirox treatment increases extracellular concentrations of enterobactin. We further hypothesized that gene deletions in the galactose metabolism or ECA synthesis pathways, in combination with ciclopirox treatment, would increase extracellular concentrations of enterobactin beyond the concentration reached by ciclopirox treatment alone. To test these hypotheses, we measured how ciclopirox affects enterobactin production of *E*. *coli* strain BW25113, with or without genes encoding proteins involved in galactose salvage or ECA synthesis, using a modified Arnow assay [42]. As this assay measures siderophores containing catechols, we were unable to distinguish fully formed enterobactin from its fragment precursors, which also can accumulate and bind iron in the growth medium [72].

**Fig 2:**
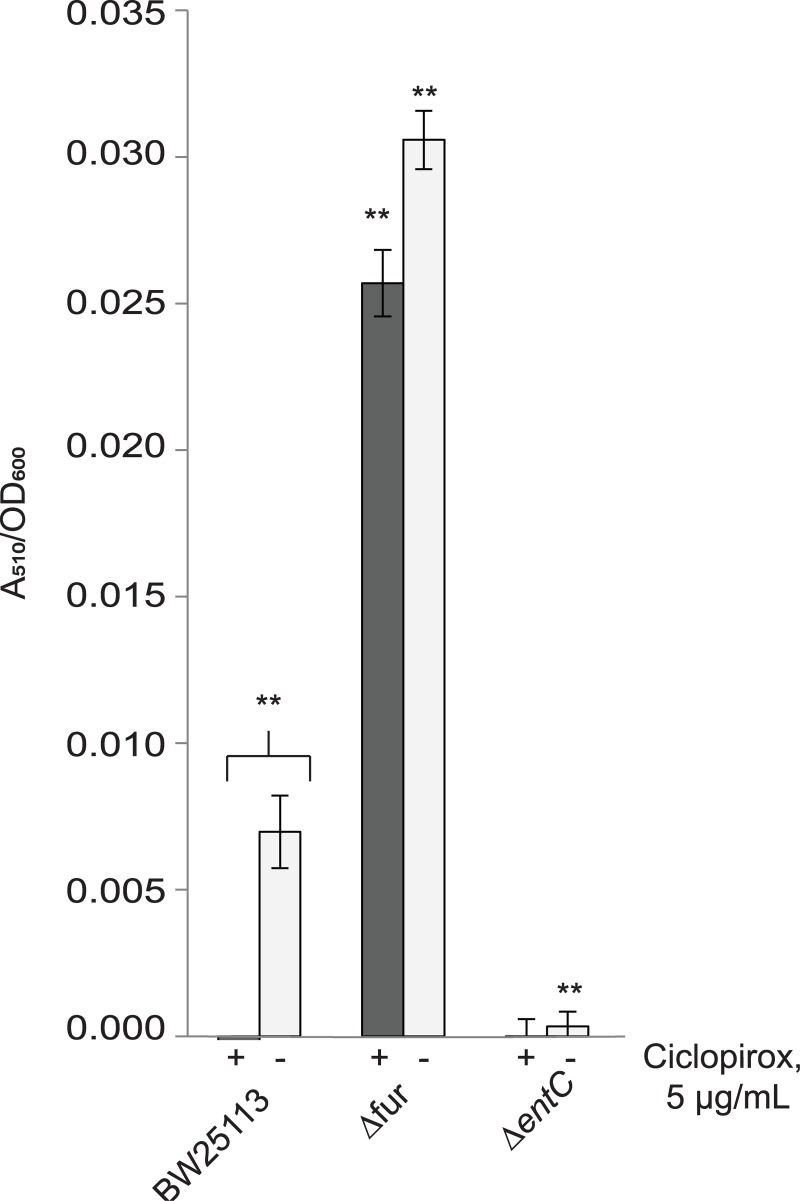
Ciclopirox stimulates enterobactin production. Catechol siderophore production by the *E*. *coli* Keio parent strain BW25113 as measured using a modified Arnow chemical assay. A student’s t-test was used to assess significance of the mutants relative to the isogenic parent strain (**, *p* <0.005).

**Fig 3:**
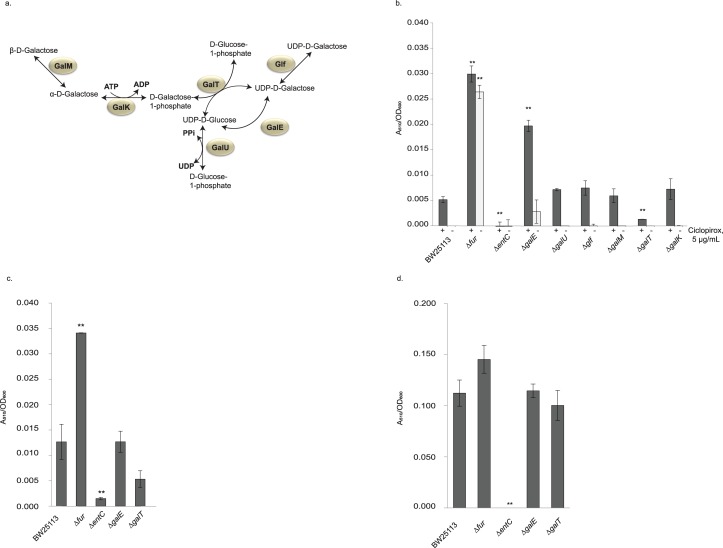
Ciclopirox-mediated enterobactin production is altered by deletion of *galE* or *galT*. (a) Schematic of galactose salvage pathway (adapted from a combination of [27,49,50]). (b) Catechol siderophore production measured using the Arnow chemical assay for the Keio parent BW25113 and select Keio mutant strains with deletions in genes encoding proteins involved in the galactose salvage pathway grown with sub-inhibitory concentrations of ciclopirox in MH medium (c) A repeat of the catechol siderophore production measurement described in (b) for a subset of strains except in the presence of sub-inhibitory concentrations of 1,10-phenanthroline. (d) A repeat of the catechol siderophore production measurement described in (c) except the bacteria were grown in low iron M9 minimal medium with no drug. A student’s t-test was used to assess significance of the mutants relative to the isogenic parent strain (**, *p* <0.005). The Δ*fur strain* and the Δ*entC* strain served as positive and negative controls, respectively.

**Fig 4:**
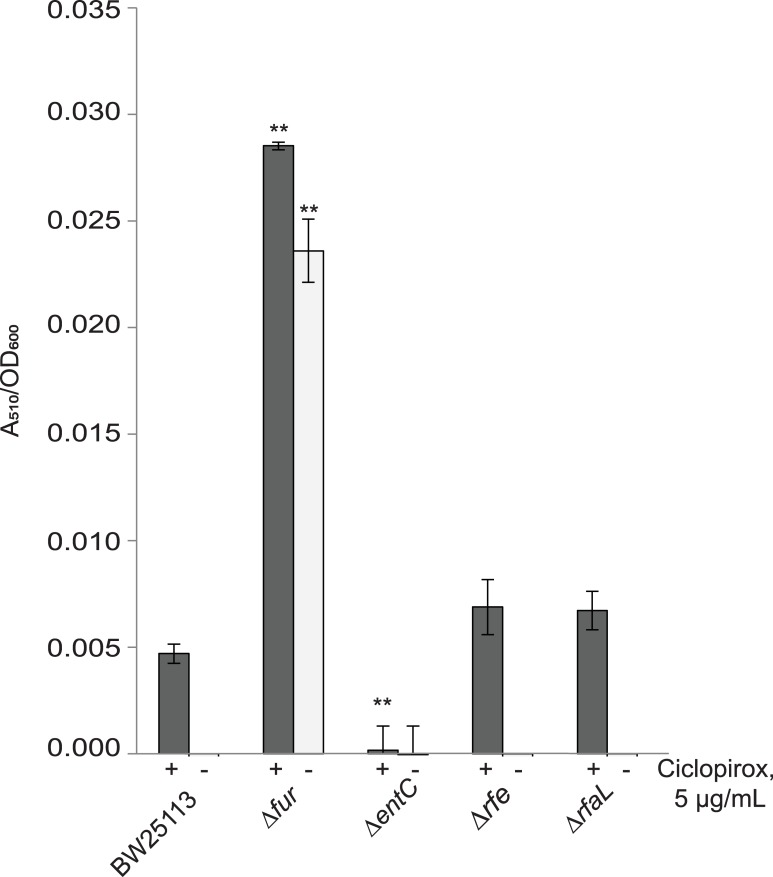
Ciclopirox-mediated enterobactin production is unaffected by disruption of the ECA synthesis pathway. Catechol siderophore production was measured as above. A Student’s t-test was used to assess significance of the mutants relative to the isogenic parent strain (**p <0.005). The Δ*fur* strain and the Δ*entC* strain served as positive and negative controls, respectively.

When the parent strain for the Keio collection (BW25113) was grown in rich growth medium, the addition of a sub-inhibitory concentration of ciclopirox indeed induced enterobactin expression (Fig 2). As negative and positive controls for this experiment, the same treatment was tested on the Δ*entC* strain, which is unable to produce enterobactin, or the Δ*fur* strain, which produces high concentrations of enterobactin because the biosynthesis machinery for enterobactin production remains active without the *Fur* repressor [65]. With or without ciclopirox in the growth medium, the Δ*fur* strain produced similar concentrations of enterobactin while Δ*entC* strain produced no enterobactin (Figs 2–4). Thus, ciclopirox stimulates enterobactin production.

We measured enterobactin production in *E*. *coli* strains without genes encoding proteins in the galactose salvage pathway (Fig 3A) [27,49]. Of six galactose salvage mutants tested, the Δ*galE* mutant strain produced more (*p* < 0.005) enterobactin than the parent strain when grown overnight with a sub-inhibitory concentration (5 μg/mL) of ciclopirox. The Δ*galT* strain produced less enterobactin than the parent (*p* < 0.005) (Fig 3B). To distinguish whether these changes were specific to ciclopirox-induced enterobactin production or were the result of iron sequestration by ciclopirox, we measured enterobactin production of these two mutant strains and their isogenic parent strain grown in either rich growth medium with a sub-inhibitory concentration of 1,10-phenanthroline or M9 minimal growth medium with no iron added (Fig 3C and 3D). Glucose was used in the M9 medium, as mutants in the galactose salvage pathway fail to grow when galactose is the sole carbon source [48].

When grown with 1,10-phenanthroline, the parent strain and the two mutant strains produced similar levels of enterobactin (Fig 3C). Because siderophore production is upregulated in low iron medium, enterobactin levels of the Δ*galT* and Δ*galE* strains were comparable to the Δ*fur* mutant and parent strain in M9 minimal growth medium. Thus, deletion of *galE* or *galT* in the galactose salvage pathway caused diametrically opposed changes in enterobactin production induced by ciclopirox while there were no changes induced by 1,10-phenanthroline or as a consequence of low iron in the growth medium, demonstrating that iron sequestration is not the cause. Therefore, the increase of ciclopirox-triggered siderophore production in these galactose salvage mutants are not a response to low iron but are a specific response to ciclopirox. These findings brought up the possibility that ciclopirox affecting a single target protein may affect these two pathways simultaneously.

To test whether disruption of ECA synthesis also affects ciclopirox-induced enterobactin production, we performed a modified Arnow Assay on ECA synthesis pathway deletion mutant strains (Fig 4). As mentioned above, the Δ*wecA* strain does not synthesize ECA and the Δ*waaL* strain is unable to attach ECA to core LPS. If ciclopirox increases siderophore concentrations through a mechanism that is also affected by the loss of ECA, we would expect to see an increase in extracellular enterobactin for strains unable to synthesis ECA. We found, however, that both ECA mutant strains produced the same concentrations of enterobactin as the parent strain. Therefore, neither the loss of ECA attached to core LPS nor the complete loss of ECA, affects ciclopirox-induced enterobactin production. There must be a different explanation, perhaps ciclopirox interacting with a downstream component, for why deletion of ECA synthesis genes contributes to a lower ciclopirox MIC.

### Ciclopirox affects bacterial swarming

In trying to understand why the ECA synthesis pathway mutants had lower ciclopirox MICs without concomitant changes in enterobactin concentrations, we examined the downstream consequences of deleting ECA synthesis genes. Although it does not destabilize LPS, disruption of ECA synthesis undermines additional functions of the cell envelope, leading to the accumulation of glycolipid intermediates and triggering cell stress response pathways, including activation of the regulation of capsular synthesis (Rcs) stress response [62,73–76]. The Rcs stress response is a signal transduction phosphorelay system that controls multiple cellular pathways, including capsule synthesis, biofilm formation, virulence, and inhibition of flagella synthesis [73]. Rcs signaling is thought to shift *E*. *coli* from a motile phenotype to a biofilm-forming phenotype, as surface contact is thought to trigger both phenotypes [73,77–79]. Nine different mutants (from 11 tested) in genes from the ECA synthesis pathway decrease swarming [80], a specific surface motility where bacteria move in “rafts” across surfaces powered by flagella. Swarming contrasts with swimming where bacteria individually move through a liquid [78]. We hypothesized that ciclopirox induces *E*. *coli* to swarm. The ECA synthesis deletion strains combined with ciclopirox treatment would be, therefore, inducing the bacteria toward different phenotypes, stressing the bacteria and potentially explaining the lower ciclopirox MICs for these deletion mutants.

To assess the effect of ciclopirox on bacterial swarming, logarithmically growing bacterial cultures of BW25113 were placed on the surface of a semi-solid "Eiken" agar, a "wet" agar thought to have lower surface tension needed for *E*. *coli* K-12 to swarm [80,81] Swarming was measured as the total area of agar surface covered by bacteria. Swarming is measured on a lower percentage agar plate (0.5%) compared to the percentage typically used for bacterial growth (1.5%), which is restrictive to swarming. No motility of strain BW25113 was observed on 1.5% agar (data not shown).

Obtaining consistent results among different experiments on 0.5% Eiken agar proved challenging because *E*. *coli* is extraordinarily sensitive to the “wetness” of the agar [82]. The majority of the time *E*. *coli* swarmed, but the swarming ranged from no swarming (round colony) to extensive swarming (S1 Fig). Despite this variability, we were able to assess the effect of a range of sub-inhibitory ciclopirox concentrations on swarming.

**Fig 5:**
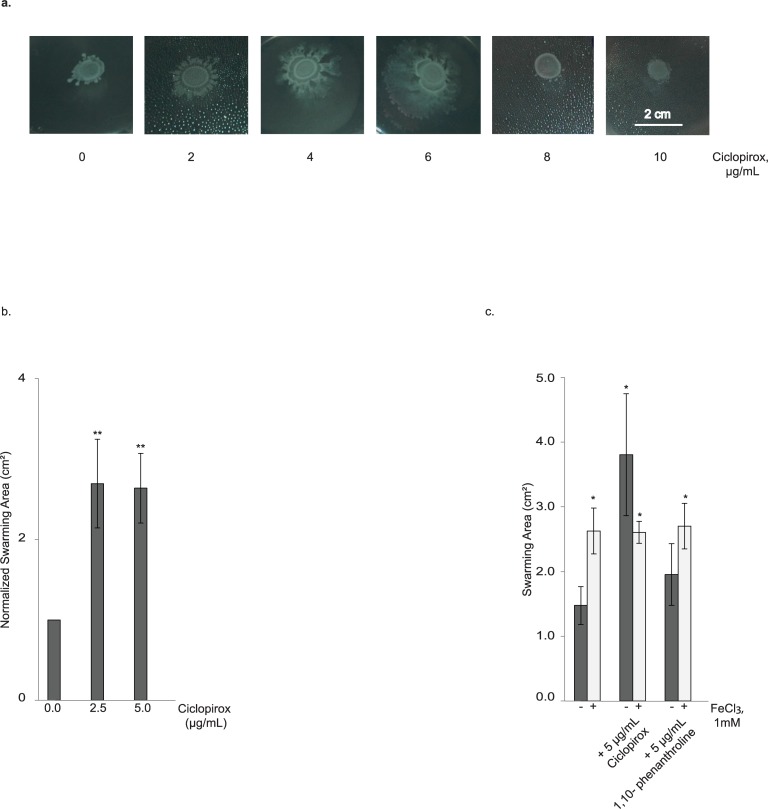
Sub-inhibitory concentrations of ciclopirox increase swarming of *E*. *coli* strain BW25113. (a) Representative data. Bacterial cultures were dotted on the surface of LB Eiken agar containing various concentrations of ciclopirox. (b) Swarming on agar containing 2.5 or 5 μg/mL ciclopirox was normalized relative to swarming on agar without ciclopirox. (c) Area of swarming motility for *E*. *coli* grown without or with 5 μg/mL ciclopirox or 5 μg/mL 1,10-phenanthroline, as well as without or with 1,000 μM FeCl_3_, to examine combinatory effects. Student’s t-tests were used to assess statistical significance (*p < 0.05; **p < 0.005).

Swarming increased with increasing sub-inhibitory ciclopirox concentrations (Fig 5A). Repeated testing revealed that the area of bacterial swarming reproducibly increased with sublethal concentrations of ciclopirox (2.5 or 5 μg/mL) and these results were statistically significant (*p* < 0.05) (Fig 5B). While we were preparing this work for publication, a study was published that concluded *E*. *coli* grown in 12.5 μg/mL of ciclopirox swarmed less and down-regulated the expression of 4/5 motility-associated genes [29]. At 10 μg/mL ciclopirox, our highest tested concentration, we also saw decreased swarming for this K12 laboratory strain. The decrease in swarming at near-MIC concentrations of ciclopirox may reflect an inhibition of cell growth; time-kill curves of the *E*. *coli* strain ATTC 25922 grown in liquid culture showed that ciclopirox is bacteriostatic at concentrations near the MIC [27].

To assess whether the increase in swarming was a consequence of iron chelation, we tested the effect of ciclopirox and iron in combination. We performed an initial test of a wide range (0, 5, 10, 50, 100, 1,000 μM, final concentrations) of supplemental iron. Swarming slightly increased at the highest concentrations (100 and 1,000 μM) (S2 Fig). *E*. *coli* strain BW25113 was dropped onto Eiken agar with or without 5 μg/mL ciclopirox and 1,000 μM supplemental iron (Fig 5C). As expected, ciclopirox increased swarming of BW25113 compared to the control conditions. In combination, however, iron caused no additional swarming. For comparison with an iron chelator, swarming was measured on Eiken agar containing 5 μg/mL 1,10-phenanthroline without or with 1,000 μM supplemental iron (Fig 5C). Compared to the control conditions, 1,10-phenanthroline had no effect on swarming. Taken altogether, these data suggest that ciclopirox-induced increases in swarming are independent of its iron chelation activity.

**Fig 6:**
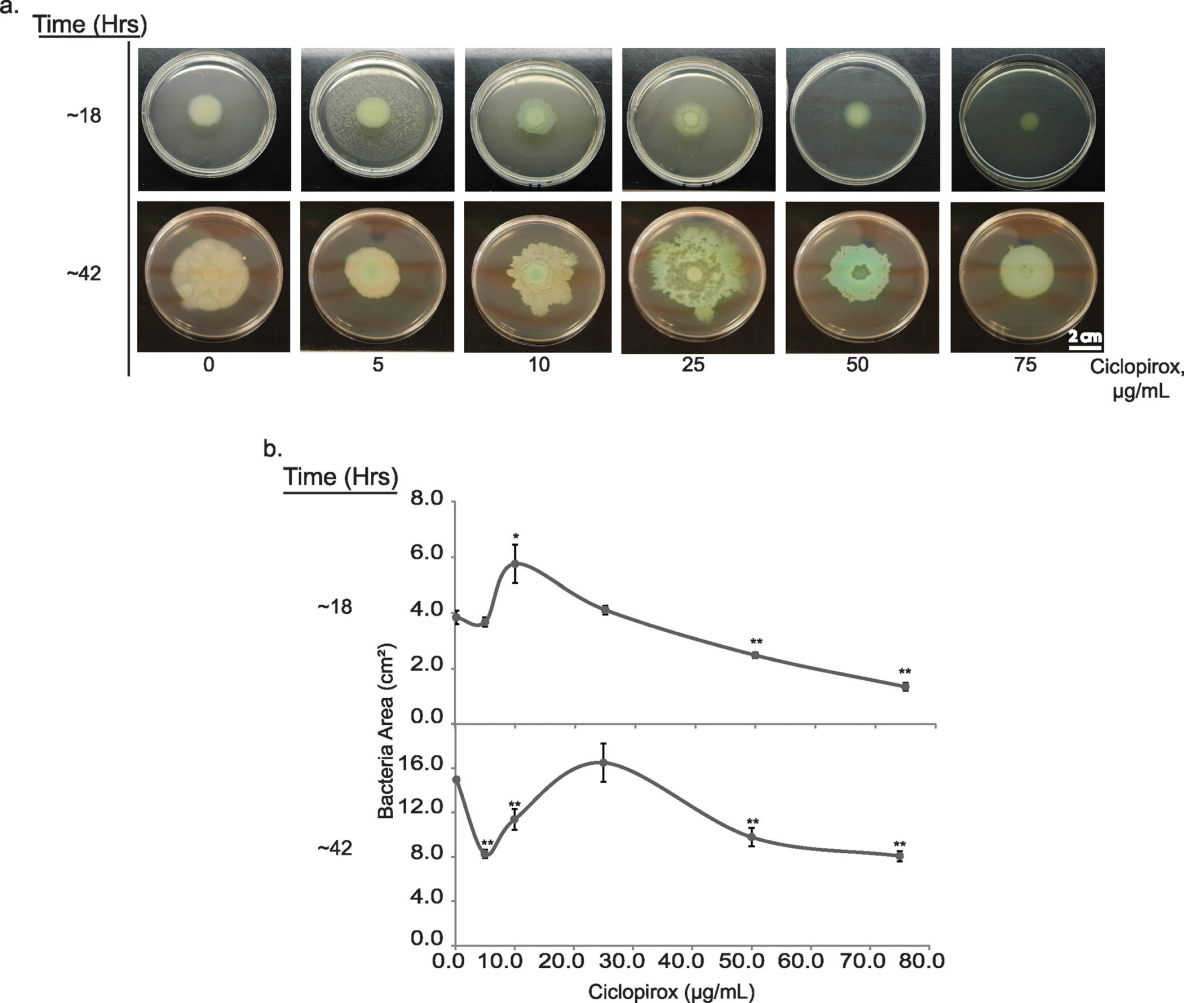
Sub-inhibitory concentrations of ciclopirox increase *P*. *aeruginosa* swarming. (a) Representative data of *P*. *aeruginosa* strain ATTC 27853 swarming after ~18 or ~42 hours. Yellow indicates pyoverdine production; bluish-green indicates pyocyanin production. (b) Quantification of swarming motility in cm^2^_._ A Student’s t-test was used to assess significance of swarming with ciclopirox compared to the absence of ciclopirox (* *p* < 0.05, **p < 0.005).

To explore the effect of ciclopirox on a bacterial species that swarms more readily than the *E*. *coli* strain BW25113, we tested *P*. *aeruginosa* strain ATCC 27853. As the ciclopirox MIC for this strain is greater than 30 μg/mL [27], we assessed a different range of ciclopirox concentrations (0, 5, 10, 25, 50, 75 μg/mL). Because *P*. *aeruginosa* produces pyoverdine, a yellowish-green siderophore, we were also able to examine siderophore production visually [27]. After 18 hours of growth on Eiken agar, there was a significant (*p* < 0.05 by Student's t-test) increase in swarming for *P*. *aeruginosa* grown in 10 μg/mL ciclopirox compared to no ciclopirox (Fig 6). In addition, all the bacteria grown on agar containing ciclopirox displayed a pale yellow indicative of pyoverdine production. After 42 hours of growth, this yellow pyoverdine became more pronounced and the bluish-green pyocyanin was also produced (Fig 6). Pyocyanin is a virulence factor and quorum sensing signaling molecule produced under low iron conditions [83]. At 25 μg/mL of ciclopirox, there was not a significant additional increase in swarming after 42 hours but over that time visible spaces formed as rafts of bacteria moved toward the edges of the plate (Fig 6A). Together, these data suggest that sub-inhibitory ciclopirox increases swarming for *P*. *aeruginosa* and induces pyoverdine and pyocyanin production.

One possible explanation for ciclopirox-induced swarming is that ciclopirox may lessen surface tension as a surfactant. Alternatively, ciclopirox may induce production of bacterial surfactants. Two different surfactant assays were performed for overnight cultures of *E*. *coli* or *P*. *aeruginosa* (S3 Fig) to test these possibilities. *P*. *aeruginosa* strain ATTC 27853 naturally produces surfactants while the BW25113 *E*. *coli* strain does not [81,84]. In one assay, a drop collapse assay, cells are pelleted and a drop of the supernatant is placed over a thin layer of mineral oil [43]. The drop “collapses” when there are surfactants present. In the other assay, an oil displacement assay, drops of bacterial supernatant are dropped into water covered by a thin layer of unrefined crude oil [44,45]. In this assay, surfactants increase the size of the oil-displaced circle as a function of surfactant concentration. Triton X-1000 was used as a positive surfactant control. Both of the expected phenotypes, a collapsed drop and increased surface oil displacement occurred from the surfactant present in the *P*. *aeruginosa* supernatant. These phenotypes were not changed by the addition of ciclopirox at several different concentrations (S3 Fig). *E*. *coli* supernatant, alone, did not produce surfactant, as indicated by no collapsed drop and no increased oil displacement. Ciclopirox had no effect on either *E*. *coli* surfactant assay. Thus, ciclopirox does not stimulate swarming by producing surfactant. As such, we considered alternate explanations for why ciclopirox may affect *E*. *coli* swarming.

### Ciclopirox-induced swarming is not mediated through Rcs phosphorelay

Disruption of the ECA synthesis pathway activates the Rcs stress response, which inhibits bacterial swarming and increases biofilm production [73,74]. As ciclopirox MICs are lowered in ECA mutants, we hypothesized that ciclopirox may inhibit activation of Rcs phosphorelay. We, therefore, compared swarming and biofilm formation for the Δ*rcsB* and Δ*rcsA* strains with increasing concentrations of ciclopirox. RcsB is the response regulator of Rcs phosphorelay, which, either through binding RcsA or dimerizing with itself, activates transcription of Rcs target genes [73]. The Δ*rcsB* strain migrates farther and more quickly on Eiken agar and the Δ*rcsA* strain swarms like the isogenic parent [85]. If ciclopirox inhibits a protein in the Rcs phosphorelay, we would expect ciclopirox to have no effect on either swarming or biofilm production in the Δ*rcsB* strain where the response regulator is absent.

**Fig 7:**
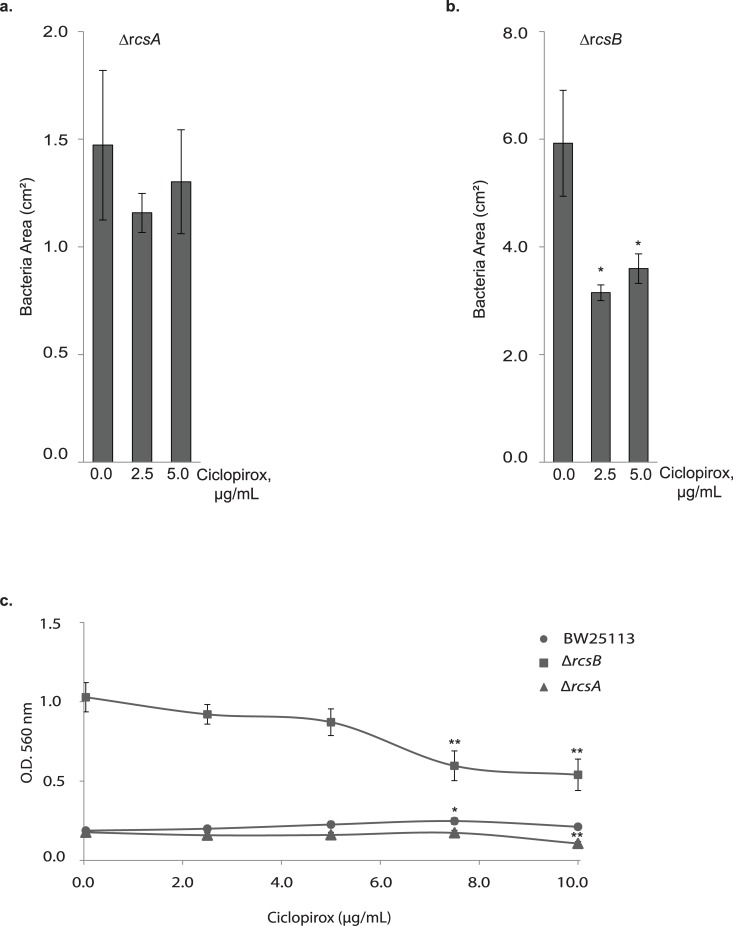
Ciclopirox does not increase swarming or biofilm production in Rcs effector deletion strains. (a) Bacteria swarming in 0, 2.5, or 5 μg/mL ciclopirox for the Δ*rcsA* strain (b) Bacteria swarming in 0, 2.5, or 5 μg/mL ciclopirox for the Δ*rcsB* strain (c) An MTT assay was used to assess absorbance at 560 nm, indicative of biofilm production, for the parent *E*. *coli* strain BW25113 and the Δ*rcsA* or Δ*rcsB* deletion mutant strains. Student’s t-tests were used to assess significance (* *p* < 0.05, ** *p* < 0.005).

We analyzed motility and biofilm production in the presence of varying concentrations of ciclopirox. The parent strain, BW25113, and the mutant strains Δ*rcsB* or Δ*rcsA* were grown in the presence of ciclopirox (0, 2.5, or 5 μg/mL) (Fig 7A and 7B). Ciclopirox-stimulated swarming did not occur in the Δ*rcsA* strain while in the Δ*rcsB* strain, ciclopirox decreased swarming (Fig 7A and 7B). Because ciclopirox has an effect on swarming in a strain lacking the RcsB response regulator, it is unlikely that ciclopirox inhibits the Rcs phosphorelay.

Because Rcs activation shifts *E*. *coli* away from a motile phenotype and toward a biofilm forming phenotype, biofilm production for each strain was measured as a function of ciclopirox concentration (0, 2.5, 5, 7.5, or 10 μg/mL). The biofilm was stained using the MTT assay, where the absorbance of formazan dye at 560 nm indicates viable cells in a biofilm (Fig 7C). Ciclopirox-induced changes to biofilm formation followed a similar pattern to ciclopirox-induced changes to bacterial swarming. BW25113 had a slight statistically significant (*p* < 0.05) increase in biofilm production at 7.5 μg/mL of ciclopirox compared to no ciclopirox and there was no change for the Δ*rcsA* mutant strain (Fig 7C). The amount of biofilm produced by Δ*rcsB* decreased with increasing concentrations of ciclopirox (Fig 7C). Thus, ciclopirox does not appear to shift bacteria towards a swarming phenotype and away from a biofilm forming phenotype in this strain.

Ciclopirox decreased both swarming motility and biofilm production for the Δ*rcsB* strain and had no effect on either phenotype of the Δ*rcsA* strain. The lack of change in either swarming or biofilm production of the Δ*rcsA* mutant could be explained by the fact that RcsA is unstable anyway, so its cellular levels are already low [74]. These data could suggest that ciclopirox-induced swarming is eliminated in strains lacking Rcs effector proteins, but the decrease in both biofilm production and bacterial swarming in the Δ*rcsB* strain reveals ciclopirox has an effect on bacteria independent of the Rcs phosphorelay. Disruption of ECA synthesis, however, induces the Rcs phosphorelay [62,76]. Perhaps, then, the downstream targets of the phosphorelay contain proteins involved in outer membrane structure, or other protein targets, with which ciclopirox interacts.

### Effect of ciclopirox on swarming of gram-negative clinical isolates

Surface motility was measured for *A*. *baumannii* strain ATTC17978, *E*. *coli* strain ATTC BAA-1743 SMS-3-5, *K*. *pneumoniae* strain ATTC 11296, and a clinical isolate previously examined in our laboratory ELZ 4486 [27], at various different concentrations of ciclopirox (0, 1.25, 2.5, 5 μg/mL). Previous testing reveals these strains are all susceptible to ciclopirox [27]. This was not a comparison of swarming motility, just surface motility, as *K*. *pneumoniae* is usually a non-flagellated bacterium, although a flagella expressing *K*. *pneumoniae* isolate was identified in 2016 [86]. *A*. *baumannii* is also considered non-motile and also does not produce flagella. Instead, *A*. *baumannii* undergoes a different surface motility most similar in appearance to swarming of *P*. *aeruginosa* [87]. Thus we do not refer to cell movement across the agar surface for non-flagellated bacteria as swarming motility, just surface motility.

Initial testing revealed that several of the clinical isolates robustly moved on the “wetter” Eiken agar (data not shown). Thus, we used 0.5% agar to reduce overall motility. There were variable responses to ciclopirox (S4 Fig). The *A*. *baumannii* ATTC17978 and the *E*. *coli* strain ATTC BAA-1743 SMS-3-5 isolates were less motile with increasing concentrations of ciclopirox, but the ELZ4486 *E*. *coli* isolate swarmed more. The *K*. *pneumoniae* ATTC 11296 isolate at 5 μg/mL of ciclopirox displayed some motility in two of three replicates (S4 Fig).

Thus, while ciclopirox-induced swarming in the BW25113 K-12 *E*. *coli* strain, it has highly varying effects on clinical isolates. From these data, it is clear that ciclopirox affects motility.

## Discussion

### Ciclopirox affects LPS

In this work, we identified multiple ways that ciclopirox affects *E*. *coli*. As detailed below, all of these ways can be explained by ciclopirox changing LPS. We had shown previously that sub-inhibitory concentrations of ciclopirox decrease O-antigen and high molecular weight LPS [27]. Others demonstrated that polymyxin B, which binds LPS, synergizes with ciclopirox [5,88]. Furthermore, the polymyxin B-ciclopirox synergy is lost in *E*. *coli* strains lacking *waaJ*, *waaL*, or *waaP*, genes that encode core LPS synthesis function [5]. The synergy is also lost in a strain lacking *galU* [5].

In *Vibrio cholera*, mutation in *galU* alters the expression of LPS core oligosaccharide but not O-antigen. This mutant leaks periplasmic proteins and is more sensitive to organic acids, hydrophobic agents, and cationic peptides than its parent strain [89]. It also has a lower polymyxin B MIC [89]. These findings suggest that the integrity of the outer membrane of the *galU* mutant is compromised. Thus, if the role of GalU *in V*. *cholera* is similar to its role in *E*. *coli*, the consequences on the structure of core LPS could explain the loss of synergy between ciclopirox and polymyxin B in the Δ*galU* mutant in *E*. *coli*.

In the *V*. *cholera* studies, deletion of *galE* did not affect expression of LPS core oligosaccharide as this species is thought to contain no galactose in its LPS [89]. In *Porphyromonas gingivalis*, however, a species with galactose in its LPS, loss of *galE* results in a change of production from high to low molecular weight LPS [90]. Like *P*. *gingivalis*, *E*. *coli* incorporates galactose into both high molecular weight LPS and O-antigen [35]. K-12 *E*. *coli*, however, do not express O-antigen, but GalE in these K12 strains nonetheless contributes to high molecular weight core LPS [27]. For *E*. *coli*, either increasing the iron concentration or overexpressing *galE* allows cells to survive ciclopirox [27]. This result, in combination with the data showing the LPS-dependence of the synergy between polymyxin B and ciclopirox, suggest that ciclopirox alters the composition of core LPS.

### Ciclopirox affects enterobactin transport

The concentration of the extracellular iron scavenger, enterobactin, increased in response to a sub-inhibitory concentration of ciclopirox in *E*. *coli* K12 strain BW25113. The most obvious explanation for this result is that ciclopirox is an iron chelator. Enterobactin, however, should outcompete ciclopirox for iron because the binding constant of Fe(III) for enterobactin is 10^52^, the tightest known siderophore iron binding affinity [91,92]. The estimated binding constant for Fe(III)-ciclopirox is expected to be similar to deferoxamine, 10^31^ [93]. Therefore, an excessive amount of enterobactin would be expected to easily outcompete ciclopirox for available iron, and, thus, prevent ciclopirox-mediated bacterial inhibition, but it does not. Additionally, enterobactin is overexpressed in the *E*. *coli* Δ*fur* mutant yet this strain has the same ciclopirox MIC as the isogenic parent. These results demonstrate that ciclopirox cannot be just an iron chelator, and therefore its effect on enterobactin production is not a consequence of iron chelation.

Ciclopirox may stimulate the release of siderophore or siderophore precursors. The Arnow Assay measures catechol siderophores, which cannot distinguish enterobactin from its precursors such as 2,3-dihydroxy-benzoic acid or its associated dihydroxybenzoylserine, both of which can bind and uptake iron [72,94]. In support of this possibility, in *C*. *albicans*, ciclopirox induces leakage of cellular constituents, albeit at higher concentrations (50 μg/mL) of ciclopirox than we used [95]. Uptake of siderophore is through the transporter FepA but the Fiu and Cir transporters transport the enterobactin precursor dihydroxybenzoylserine into the cell [94]. We found that the Δ*fepA* strain but not the Δ*fiu* strain had a lower ciclopirox MIC than the parental strain (S1 Table), suggesting that the transport of enterobactin is specifically important for sensitivity to ciclopirox. As we are not using a high concentration of ciclopirox, where constituent leakage could be induced, and deletion of the enterobactin, not the enterobactin precursor, outer membrane transporter has a lower ciclopirox MIC, it is unlikely that ciclopirox is increasing release of enterobactin precursors at the concentrations of ciclopirox tested.

It is possible that ciclopirox-induced changes in LPS expression may decrease siderophore uptake. Modifications of cell surface glycolipids affect siderophore uptake in *Vibrio anguillarum* [71]. The disruption of either of two different genes required for production of the O-antigen side chain component, dTDP-rhamnose, results not only in loss of high molecular weight O-antigen but also reduced expression of the outer membrane receptor FatA [71]. As a consequence of the loss of FatA, anguibactin, the siderophore of *V*. *anguillarum*, is overexpressed independently of iron concentration in the growth medium [71]. It is unclear exactly how the loss of either of these genes results in the loss of FatA [71], but the data show that changes in surface glycolipids can have consequences for siderophore transport.

In *E*. *coli*, the O-antigen length regulator protein, FepE, is required for synthesis of very long chain O-antigen [54]. *E*. *coli* with an insertion mutation in the *fepE* gene are able to synthesize enterobactin but are deficient in enterobactin transport. The Δ*fepE* strain fails to grow in iron-depleted growth medium, even with the addition of purified enterobactin [64]. Thus, synthesis of very long chain O-antigen can affect enterobactin transport. In *Salmonella typhimurium*, the density of very long chain O-antigen increases under low iron conditions, suggesting that low iron may signal very long chain O-antigen expression [96]. Based on these data it seems that the expression of very long chain polysaccharides is important for enterobactin transport. Because ciclopirox induces changes to the bacterial LPS (in particular high molecular weight LPS) as well as siderophore transport, the ciclopirox-induced changes in LPS could prevent uptake of enterobactin.

In the context of a treatment option for bacterial infections, it would be preferable for ciclopirox to prevent siderophore transport across the bacterial membrane and not target a single specific siderophore pathway, as there are hundreds of siderophores, but all iron-bound siderophores must cross the bacterial membrane to reenter the cell. As our data revealed a ciclopirox-induced increase in enterobactin in *E*. *coli* and pyoverdine in *P*. *aeruginosa* ((Fig 6.) and [27]), ciclopirox may affect siderophore production via affecting the membrane in different species. Targeting the bacterial membrane with ciclopirox could prevent uptake of siderophores across many different bacteria.

### Ciclopirox affects surface motility

Bacterial swarming is highly dependent on cell density, growth conditions, and surface moistness [97]. Robust swarmers capable of moving on “hard” agar undergo a swarming-specific transcriptional program to change structure and become hyperflagellated; temperate swarmers, such as *E*. *coli*, require “soft” agar to swarm and do not become hyperflagellated. [82]. Instead, in *E*. *coli*, swarming depends upon surface-colony hydration, which is controlled by the ability of the cells to switch motor direction (clockwise or counterclockwise) of the flagella [82]. Attracting water from the agar gel to the surface is a challenge for bacteria and can explain the sensitivity to the “wetness” of the plate [81]. It is thought that flagella rotation during swarming hydrates the colony either by secreting water, by drawing water from the agar through turbulence, or by stripping LPS off of the bacterial outer membrane to use as an osmolyte [82].

The surface motility of non-flagellated bacteria has not been fully explained. Different strains of *A*. *baumannii* display different patterns of motility, with varying contributions of twitching motility and multiple additional factors [87,98,99]. LPS is a contributing factor, as mutants defective in LPS core synthesis are defective in motility [99].

As LPS is a common factor in bacterial swarming and surface motility across species, ciclopirox-induced changes in LPS may account for the different swarming phenotypes of the various tested bacteria in response to ciclopirox. Indeed, in addition to LPS and ECA being required for *E*. *coli* swarming by serving as cell surface wetting agents, alterations to the inner core LPS are thought to disrupt function and assembly of the flagella [80]. Thus, loss of high molecular weight LPS and possible changes in LPS composition caused by ciclopirox could explain the reduced swarming of some *E*. *coli* clinical isolates.

A ciclopirox-induced increase in local hydration through stripping of LPS could potentially explain the increased swarming and surface motility of the BW25113 strain and some clinical isolates. *E*. *coli* also double in size during swarming, thereby doubling the number of flagella per cell [82]. The average BW25113 strain cell length increases from 2.8 to 3.4 μM when treated with 12.5 μg/mL ciclopirox [29]. At this ciclopirox concentration, however, BW25113 swarming decreases [29].

### Ciclopirox is not just an iron chelator

As ciclopirox is an iron chelator, it is generally expected that its mechanism of inhibition is through general iron binding. Quite unexpectedly, our data indicate that ciclopirox actually restricts iron utilization through two different methods, direct chelation of free iron and indirect prevention of siderophore uptake. In addition, the efficacy of this drug has repeatedly been shown to extend beyond just the binding of free iron. In the treatment of HIV infection, for example, the structure of ciclopirox allows it to bind iron specifically in the active site of a protein needed for synthesis of a rare amino acid required for a single known protein [26,33]. In the case of *E*. *coli*, we have identified several ciclopirox-mediated effects that do not appear to be dependent upon free iron binding, but may be dependent upon how the structure of the glycolipids on the bacterial outer membrane change in response to ciclopirox. There is also the possibility of other specific pathways targeted by ciclopirox. While we were preparing this document for publication, a paper was published on mRNA profiling of the BW25113 *E*. *coli* strain grown with sublethal concentrations of ciclopirox. In that work, they identified 514 differentially expressed genes, including 123 involved in metabolite transport and down-regulation of an Rcs protein gene (*rcsD)* [29]. They also identified the involvement of glutamate-dependent acid resistance genes and the global transcriptional silencer encoded by the gene *hns* in ciclopirox susceptibility in *E*. *coli* [29].

Six of the differentially expressed genes were in our 21 genes of interest from our MIC testing [29]. Four ECA synthesis genes (*rffH*, *wecA*, *wecF*, and *wzzE*) were down-regulated and two galactose salvage genes (*galK* and *galM*) were up-regulated. These data agree with our observations that galactose salvage is important for ciclopirox and also provides evidence that ECA expression is decreased in the presence of ciclopirox. The fact that no enterobactin synthesis genes were differentially expressed supports our idea that ciclopirox interferes with enterobactin transport indirectly through modification of the bacterial membrane.

The differentially expressed genes also included five other genes in our initial collection of 96 mutant strains, which did not have a lower ciclopirox MIC compared to the isogenic parent strain. These genes include up-regulation of *basR*, which encodes part of a two-component iron and zinc sensing transcription regulator that regulates genes associated with modification of membrane structure, modulation of membrane function, and modulation of stress response cell functions [100]. Additional differentially expressed genes included up-regulation of *fnr*, which encodes the bacterial anaerobic regulator [65], up-regulation of *perR*, which encodes a peroxide stress response regulator [101], up-regulation of *phoB*, which encodes the inorganic phosphate response regulator [102], and down-regulation of *wzxE*, which encodes the ECA flippase [54]. Besides *wzxE*, we are interested in exploring why deletion of these genes encoding regulatory proteins did not lower ciclopirox MICs even if they are up-regulated in response to the drug.

While we cannot conclude that these different bacterial effects result from ciclopirox blocking a single target, we can conclude from our data that ciclopirox affects siderophore transport and swarming independently of iron chelation.

## Supporting information

S1 Fig Variations in swarming of the *E*. *coli* strain BW25113. Representative data showing variation in swarming of *E*. *coli* strain BW25113 inoculated on LB Eiken agar [80].
(EPS)

S2 Fig High concentrations of supplemental iron increase *E*. *coli* swarming. Representative data showing swarming of BW25113 *E*. *coli* with either no additional FeCl_3_ or with the indicated concentrations of FeCl_3_.
(TIFF)

S3 Fig Ciclopirox does not induce surfactant production in *E*. *coli* or *P*. *aeruginosa*. *E*. *coli* strain BW25113 or *P*. *aeruginosa* strain ATTC 27853 was grown overnight with either no drug or with the indicated concentrations of ciclopirox. Cells were pelleted with centrifugation and supernatants were spotted on the surface of mineral oil (Drop Collapse) or onto a thin layer of crude oil over water (Oil Displacement). Collapsed drops indicate the presence of surfactants. The size of the “hole” in the crude oil indicates the amount of surfactant present. Each square is a 1 cm grid.
(EPS)

S4 Fig Ciclopirox affects surface motility of gram-negative clinical isolates. Representative data (of at least three and sometimes six replicates) showing surface motility of *A*. *baumannii* strain ATTC17978, *E*. *coli* strain ATTC BAA-1743 SMS-3-5, *K*. *pneumoniae* strain ATTC 11296, and *E*. *coli* isolate ELZ4486 under increasing concentrations of ciclopirox. The different results for *K*. *pneumoniae* at 5 μg/mL ciclopirox are shown. These experiments were done as above except on regular (0.5%) LB agar with 0.5% supplemental glucose.
(TIFF)

S1 Table Ciclopirox and 1,10-phenanthroline MICs for *E*.*coli* strains. (PDF)

S2 Table Ciprofloxacin and ampicillin MICs for select *E*. *coli* strains. (PDF)
