# Supplementary figures and images for: Sugar and iron: Toward understanding the antibacterial effect of ciclopirox in Escherichia coli
Source: PLoS One. 2019 Jan 11;14(1):e0210547. doi: 10.1371/journal.pone.0210547 (PMC6329577; doi:10.1371/journal.pone.0210547)

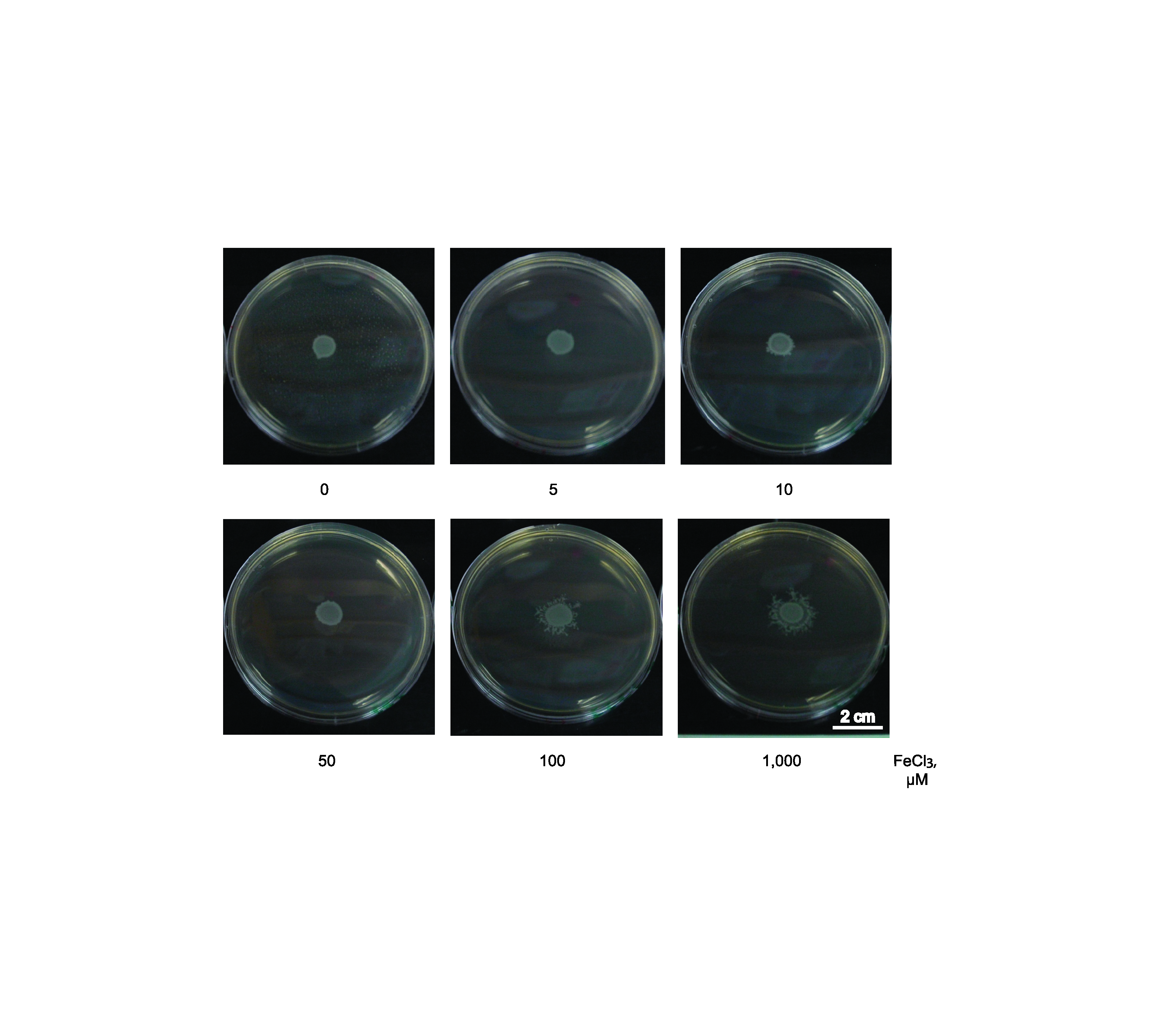

Supplement: S2 Fig — Representative data showing swarming of BW25113 E. coli with either no additional FeCl3 or with the indicated concentrations of FeCl3. (TIFF) [file pone.0210547.s002.tiff]

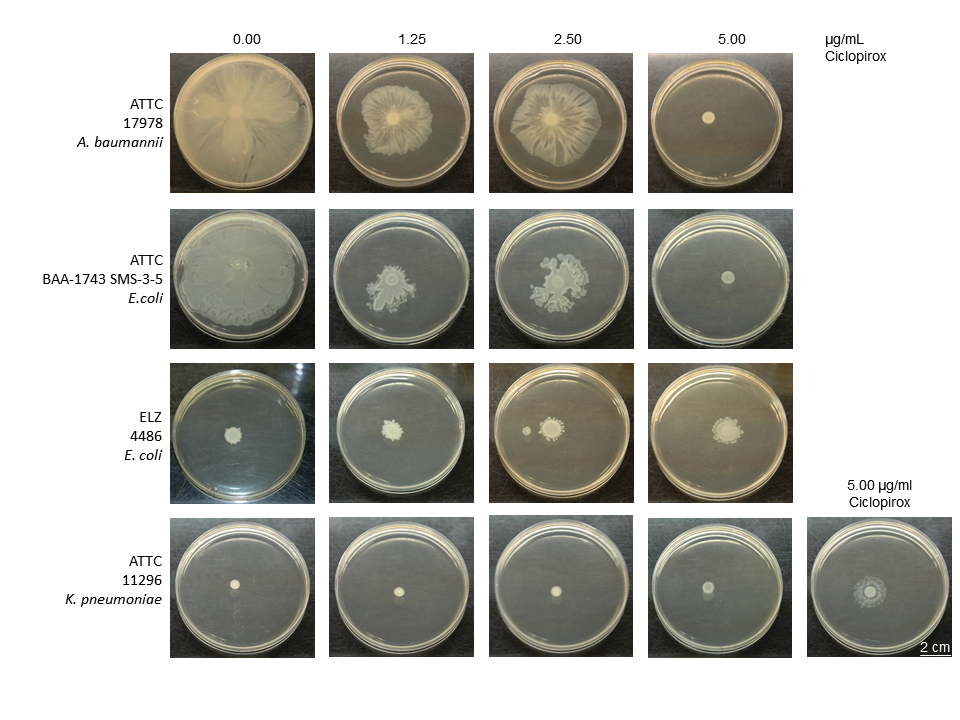

Supplement: S4 Fig — Representative data (of at least three and sometimes six replicates) showing surface motility of A. baumannii strain ATTC17978, E. coli strain ATTC BAA-1743 SMS-3-5, K. pneumoniae strain ATTC 11296, and E. coli isolate ELZ4486 under increasing concentrations of ciclopirox. The different results for K. pneumoniae at 5 μg/mL ciclopirox are shown. These experiments were done as above except on regular (0.5%) LB agar with 0.5% supplemental glucose. (TIFF) [file pone.0210547.s004.tiff]
