# Supplementary material for: Sugar and iron: Toward understanding the antibacterial effect of ciclopirox in Escherichia coli
Source: PLoS One. 2019 Jan 11;14(1):e0210547. doi: 10.1371/journal.pone.0210547 (PMC6329577; doi:10.1371/journal.pone.0210547)
Supplement: S1 Table — (PDF) [file pone.0210547.s005.pdf]

S1 Table. Ciclopirox and 1,10-phenanthroline MICs for *E.coli* strains

Sugar Metabolism						
**Gene (Synonyms[1])**	**Gene Product**	**Ciclopirox MIC Range (µg/mL)**	**Ciclopirox MIC (µg/mL)[2]**	**1,10-phenanthroline MIC Range (µg/mL)**	**1,10-phenanthroline MIC (µg/mL)[2]**	**Increased Antibiotic Susceptibility to[4]**
BW25113 (Isogenic Parent Strain)		11-18	14.2 ± 1.7	9-16	11.9 ± 1.8	None
galE (*galD*)	UDP-glucose 4-epimerase	10-15	12.3 ± 2.1	ND[3]	ND	None
galF (*wcaN, yefG*)	UTP-glucose-1-phosphate uridylyltransferase	13-15	14.0 ± 1.4	ND	ND	None
galK (*galA*)	Galactokinase	11-13	12.0 ± 0.8**	9-13	10.3 ± 2.3	None
galM	Galactose-1-epimerase	12-13	12.5 ± 0.7**	8-14	12.2 ± 3.2	None
galP (*Pgal*)	Galactose: H+ symporter	10-12	10.6 ± 0.9**	6-14	10.4 ± 4.0	None
galR (*Rgal*)	Repressor of *galETK* operon	10-15	11.9 ± 2.0	ND	ND	None
galS (*mglD*)	GalS DNA-binding transcriptional dual regulator	10-13	11.3 ± 1.5**	14	14.0 ± 0.0**	None
galT (*galB*)	Galactose-1-phosphate uridylyltransferase	10-13	11.3 ± 1.5	ND	ND	None
galU (*verA*)	UTP-glucose-1-phosphate uridylyltransferase	7-13	9.6 ± 1.6**	5-10	6.7 ± 2.1**	None
glf (*yefE*)	UDP-galactopyranose mutase	12-13	12.9 ± 0.6	8-13	10.0 ± 1.9	None
pgi	Glucose-6-phosphate isomerase	11-13	12.3 ± 1.2	ND	ND	None
pgm (*blu, pgmA*)	Phosphoglucomutase	13-18	14.7 ± 2.9	ND	ND	None
ugd (*yefA, udg, pmrE, pagA*)	UDP-glucose 6-dehydrogenase	5-12	10.6 ± 2.4**	13-15	14.0 ± 0.9	None
zwf	Glucose 6-phosphate dehydrogenase	14-15	14.6 ± 0.5	9	9.0 ± 0.0**	None
LPS Synthesis						
**Gene (Synonyms[1])**	**Gene Product**	**Ciclopirox MIC Range (µg/mL)**	**Ciclopirox MIC (µg/mL)[2]**	**1,10-phenanthroline MIC Range (µg/mL)**	**1,10-phenanthroline MIC (µg/mL)[2]**	**Increased Antibiotic Susceptibility to[3]**
fepE	Ferrienterobactin transport, membrane protein; regulator of length of O-antigen component of lipopolysaccharide chains	14-18	16.3 ± 1.9	ND	ND	None
gtrB (*yfdH*)	Bactoprenyl glucosyl transferase	12-18	14.0 ± 3.1	ND	ND	None
lptB (*yhbG*)	Lipopolysaccharide export ABC transporter ATP-binding protein	9-18	13.1 ± 4.1	ND	ND	None
rfbA (*rmlA, som*)	dTDP-glucose pyrophosphorylase	13-18	14.7 ± 1.9	ND	ND	None
rfbB (*rmlB, som*)	dTDP-glucose 4,6-dehydratase	13-15	14.2 ± 1.0	ND	ND	None
rfbC (*rfbD, rmlC*)	dTDP-4-dehydrorhamnose 3,5-epimerase	13-18	14.7 ± 1.9	ND	ND	None

rfbD (*rmlD*)	dTDP-4-dehydrorhamnose reductase	13-15	14.3 ± .8	ND	ND	None
rfbX (*wzx, wzxB*)	O-antigen flippase	13-18	15.0 ± 1.7	ND	ND	None
rfc (*wbbH, yefF)*	O-antigen polymerase	14-15	14.3 ± 0.5	ND	ND	None
rffG (*fcnA, nfrC*)	dTDP-glucose 4,6-dehydratase 2	12-14	13.2 ± 0.8	ND	ND	None
rffH (*yifG*)	dTDP-glucose pyrophosphorylase	12-13	12.5 ± 0.5**	11-13	11.7 ± 1.2	None
waaB (*rfaB, lps, syn*)	UDP-D-galactose: (glucosyl)lipopolysaccharide-1,6-D-galactosyltransferase	10	10.0 ± 0.0**	10-12	10.4 ± 0.9	None
waaC (rf*aC, rfa-2, yibC*)	ADP-heptose: LPS heptosyltransferase I	7-10	9.0 ± 1.2**	8	8.0 ± 0.0**	CHL, CST, ERY, FUS, GEN, MTR, NEO, NIT, RIF, SPT, SFX, TOB, TRI, VAN
waaD (*rfaD, hldD, htrM, nbsB*)	ADP-L-glycero-D-mannoheptose-6-epimerase	12-13	12.8 ± 0.5 **	13-14	13.3 ± 0.6	CHL, CST, ERY, FUS, GEN, MTR, NEO, RIF, SPT, STR, TOB, TRI
waaF (*rfaF*)	ADP-heptose: LPS heptosyltransferase II	11-12	12.0 ± 0.9	ND	ND	None
waaG (*rfaG*)	Lipopolysaccharide glucosyltransferase I	10-13	11.4 ± 1.7	ND	ND	CHL, CST, GEN, MTR, NEO, NIT, RIF, TOB,TRI
waaI (*rfaI, waaO*)	UDP-D-glucose: (glucosyl) LPS α-1,3-glucosyltransferase	9-10	9.2 ± 0.4**	8-11	9.0 ± 1.7	None
waaJ (*rfaJ, waaR*)	UDP-glucose: (glucosyl) LPS α-1,2-glucosyltransferase	13-14	13.3 ± 0.6	ND	ND	None
waaL (*rfaL*)	O-antigen ligase	12-15	13.3 ± 1.6	ND	ND	None
waaP (*rfaP*)	Lipopolysaccharide core heptose (I) kinase	12-15	14.0 ± 1.7	ND	ND	GEN, NEO, NIT, TOB
waaQ (*rfaQ*)	Lipopolysaccharide core heptosyltransferase III	7-9	8.0 ± 0.7**	8-9	8.3 ± 0.6**	RIF, TOB
waaS (*rfaS*)	Lipopolysaccharide core biosynthesis protein	13-15	14.3 ± 1.2	ND	ND	None
waaY (*rfaY*)	Lipopolysaccharide core heptose (II) kinase	11-13	12.5 ± 1.3	ND	ND	GEN, NIT, TRI
waaZ (*rfaZ*)	Protein involved in Kdo III attachment during lipopolysaccharide core biosynthesis	13-14	13.3 ± 0.6	ND	ND	None
wecA (*rfe*)	Undecaprenyl-phosphate α-N-acetylglucosaminyl transferase	10-13	11.4 ± 1.0**	9-14	10.9 ± 2.4	None
wecB (*rffE, yifF, nfrC, mnaA*)	UDP-N-acetylglucosamine 2-epimerase	9-12	10.8 ± 1.1**	10-11	10.3 ± 0.3**	None
wecC (*rffD, mnaB*)	UDP-N-acetyl-D-mannosamine dehydrogenase	10-12	11.0 ± 0.9**	10-11	10.3 ± 0.3**	None
wecD (*rff, rffC, yifH*)	dTDP-fucosamine acetyltransferase	12-16	13.1 ± 1.5	ND	ND	None

wecE (*rffA, fcnA, yifI*)	dTDP-4-dehydro-6-deoxy-D-glucose transaminase	11-12	11.2 ± 0.4**	11-12	11.7 ± 0.6	NEO, TOB
wecF (*yifM, rffT*)	4-acetamido-4,6-dideoxy-D-galactose transferase	10-12	11.0 ± 0.9**	11-13	12.0 ± 1.0	None
wecG (*rff, rffM*)	UDP-N-acetyl-D-mannosaminuronic acid transferase	10-11	10.8 ± 0.4**	11-12	11.7 ± 0.6	None
wzxC (*wzx*)	colanic acid translocase	9-18	12.3 ± 4.0	ND	ND	None
wzxE (*yifJ*)	lipid III flippase	10-15	12.2 ± 2.2	ND	ND	None
wzzB (*rol, cld*)	regulator of length of O-antigen component of lipopolysaccharide chains	13	13.0 ± 0.0**	10-13	11.8 ± 1.5	None
wzzE (*metNSt, yifC*)	Enterobacterial common antigen polysaccharide chain length modulation protein	9-10	9.5 ± 0.6**	10-12	11.0 ± 1.2	None
Iron Transport						
**Gene (Synonyms[1])**	**Gene Product**	**Ciclopirox MIC Range (µg/mL)**	**Ciclopirox MIC (µg/mL)[2]**	**1,10-phenanthroline MIC Range (µg/mL)**	**1,10-phenanthroline MIC (µg/mL)[2]**	**Increased Antibiotic Susceptibility to[3]**
entA	2,3-dihydro-2,3-dihydroxybenzoate dehydrogenase	6-9	7.3 ± 1.3**	11-13	12.3 ± 1.2	None
entB (*entG*)	2,3-dihydro-2,3-dihydroxybenzoate synthase	7-9	7.9 ± 0.8**	9-10	9.5 ± 0.6**	None
entC (*fepF*)	Isochorismate synthase 1	6-9	8.5 ± 1.0**	9-10	9.5 ± 0.6**	None
entE	2,3-dihydroxybenzoate-AMP ligase	7-9	8.0 ± 0.8**	9-10	9.5 ± 0.6**	None
entF	Apo-serine activating enzyme	7-9	7.9 ± 0.8**	9-10	9.5 ± 0.6**	None
entS (*ybdA*)	Enterobactin efflux transporter EntS	10-12	10.9 ± 0.9**	12-14	13.0 ± 1.0	None
exbB	TonB energy transducing system ExbB subunit	11-12	11.8 ± 0.3**	7-8	7.5 ± 0.6**	None
exbD	TonB energy transducing system ExbD subunit	11-12	11.7 ± 0.5**	7	7.0 ± 0.0**	RIF
fecA	Ferric citrate outer membrane porin	14-15	14.2 ± 0.3	ND	ND	None
fecB	Ferric citrate ABC transporter periplasmic binding protein	13-14	13.7 ± 0.5	ND	ND	None
fecC	Ferric citrate ABC transporter membrane subunit	13-15	13.8 ± 0.7	ND	ND	None
fecD	Ferric citrate ABC transporter membrane subunit	13-14	13.7 ± 0.5	ND	ND	None
fecE	Ferric citrate ABC transporter ATP binding subunit	14-15	14.8 ± 0.3**	ND	ND	None
fepA (*cbr, cbt, fep, feuB*)	Ferric enterobactin, colicin B, colicin D outer membrane porin	8-9	8.8 ± 0.5**	12-13	12.5 ± 0.6	None
fepB	Ferric enterobactin ABC transporter periplasmic binding	7-9	8.6 ± 0.7**	10-11	10.5 ± 0.6**	NEO, NIT, RIF

	protein					
fepC	Ferric enterobactin ABC transporter ATP binding subunit	7-11	8.9 ± 1.1**	10-11	10.5 ± 0.6**	CIP, NEO, NIT, RIF, STR, VAN
fepD	Ferric enterobactin ABC transporter membrane subunit	7-9	8.5 ± 0.9**	11-12	11.5 ± 0.6	None
fepG	Ferric enterobactin ABC transporter - membrane subunit	7-9	8.6 ± 0.7**	10-11	10.3 ± 0.5**	None
fes	Enterochelin esterase	7-11	9.0 ± 1.5**	5-7	6.3 ± 1.2**	None
fetA (*ybbl*)	ABC transporter with a role in iron homeostasis ATP-binding subunit	11-18	14.0 ± 3.1	ND	ND	None
fetB (*ybbM*)	ABC transporter with a role in iron homeostasis membrane subunit	10-12	11.0 ± 0.8**	11-12	11.5 ± 0.6	None
fhuA (*T1, T5rec, tonA*)	Ferrichrome outer membrane transporter	14-18	16.1 ± 1.8	ND	ND	None
fhuB	Iron (III) hydroxamate ABC transporter membrane subunit	14-20	16.3 ± 2.9	ND	ND	None
fhuC	Iron (III) hydroxamate ABC transporter ATP binding subunit	14-20	15.8 ± 2.1	ND	ND	None
fhuD	Iron (III) hydroxamate ABC transporter periplasmic binding protein	15-18	16.5 ± 1.6	ND	ND	None
fhuE	Ferric coprogen outer membrane porin FhuE	13-15	14.5 ± 0.8	ND	ND	None
fiu (*ybiL*)	Putative outer membrane receptor for iron transport	14-15	14.7 ± 0.5	ND	ND	None
fur	Fur transcriptional dual regulator	10-18	14.9 ± 3.3	ND	ND	ERY, FUS, MTR, NIT, RIF, SFX, TRI, VAN
tolC (*weeA, colE1-i, mtcB, mukA, refI, toc*)	TolC outer membrane channel	13-18	15.0 ± 2.2	ND	ND	AMP, CHL, CIP, ENX, ERY, FOX, FUS, GEN, MTR, NEO, NIT, RAD, RIF, SFX, SPT, STR, TET, TOB, TRI
tonB (*T1rec, exbA*)	TonB energy transducing system TonB subunit	6-9	8.3 ± 1.2**	6	6.0 ± 0.0**	AMP, RIF
Cellular Regulatory Sensors						
**Gene (Synonyms[1])**	**Gene Product**	**Ciclopirox MIC Range (µg/mL)**	**Ciclopirox MIC (µg/mL)[2]**	**1,10-phenanthroline MIC Range (µg/mL)**	**1,10-phenanthroline MIC (µg/mL)[2]**	**Increased Antibiotic Susceptibility to[3]**
basR (*pmrA*)	BasR transcriptional regulator	14-15	14.7 ± 0.5	ND	ND	None
basS (*pmrB*)	BasS sensory histidine kinase	14-18	16.7 ± 2.1	ND	ND	None
fnr (*frdB, nirA, nirR, ossA, oxrA*)	Fnr DNA-binding transcriptional dual regulator	15-18	16.5 ± 1.6**	ND	ND	None
iscR (*yfhP*)	IscR DNA-binding transcriptional dual	11-13	12.0 ± 1.1**	8-9	8.4 ± 0.5**	None

	regulator					
norR (*ygaA*)	NorR DNA-binding transcriptional dual regulator	14-18	16.7 ± 2.1	ND	ND	None
norW (*ygaL, ygbD*)	NADH: flavorubredoxin reductase	14-18	16.3 ± 1.9	ND	ND	None
perR	PerR transcriptional regulator	13-18	14.3 ± 2.0	ND	ND	None
phoB (*phoRc, phoT*)	PhoB transcriptional dual regulator	12-18	15.0 ± 2.1	ND	ND	None
phoP	PhoP transcriptional regulator	10-13	11.3 ± 1.0**	8-12	9.9 ± 2.2	AMP, CHL, CHL, FOX, RAD, TET
phoQ	PhoQ sensory histidine kinase	9-11	10.2 ± 0.8**	8-11	8.8 ± 1.5	None
phoR (*R1pho, phoR1, nmpB*)	PhoR sensory histidine kinase	13-15	14.2 ± 1.1	ND	ND	None
soxR (*marC*)	SoxR DNA-binding transcriptional dual regulator	14-15	14.7 ± 0.5	ND	ND	None
soxS	SoxS DNA-binding transcriptional dual regulator	13-14	13.2 ± 0.4**	9-11	10.0 ± 1.2	None
Elongation Factor P						
**Gene (Synonyms[1])**	**Gene Product**	**Ciclopirox MIC Range (µg/mL)**	**Ciclopirox MIC (µg/mL)[2]**	**1,10-phenanthroline MIC Range (µg/mL)**	**1,10-phenanthroline MIC (µg/mL)[2]**	**Increased Antibiotic Susceptibility to[3]**
efp	Elongation factor (EF-P)	12-13	12.8 ± 0.5**	10-12	11.3 ± 1.0	None
epmA (*genX, poxA, yjeA*)	EF-P-lysine lysyltransferase	12-13	12.8 ± 0.5**	11-15	13.0 ± 2.3	None
epmB (*yjeK*)	lysine 2,3-aminomutase	9-18	12.8 ± 4.1	ND	ND	None
epmC (*yfcM*)	EF-P-Lys34 hydroxylase	11-12	11.8 ± 0.5**	9	9.0 ± 0.0**	None

[1]Gene Synonyms from EcoliWiki (http://ecoliwiki.net/colipedia/index.php?title=Category:Gene_List:MG1655&pageuntil=aspS%3AGene), and EcoCyc (https://ecocyc.org/)

[2]ND = Not Done

[3]** $p < 0.005$ compared to isogenic parent *E. coli* strain BW25113 (ciclopirox MIC = 14.2 ± 1.7; 1,10-phenanthroline MIC = 11.9 ± 1.8)

[4]Adapted from [47] AMP, Ampicillin; ATM, Azetreonam; FOX, Cefoxitin; RAD, Cephradine; CHL, Chloramphenicol; CIP, Ciprofloxacin; CST, Colistin; ENX, Enoxacin; ERY, Erythromycin; FUS, Fusidic acid; GEN, Gentamicin; MTR, Metronidazole; NEO, Neomycin; NIT, Nitrofurantoin; RIF, Rifampin; SPT, Spectinomycin; STR, Streptomycin; SFX, Sulfamethoxazole; TET, Tetracycline; TOB, Tobramycin; TRI, Triclosan; VAN, Vancomycin
