# Supplementary material for: Sugar and iron: Toward understanding the antibacterial effect of ciclopirox in Escherichia coli
Source: PLoS One. 2019 Jan 11;14(1):e0210547. doi: 10.1371/journal.pone.0210547 (PMC6329577; doi:10.1371/journal.pone.0210547)
Supplement: S2 Table — (PDF) [file pone.0210547.s006.pdf]

S2 Table. Ciprofloxacin and ampicillin MICs for select *E. coli* strains

Gene (Synonyms[1])	Gene Product	Ciprofloxacin MIC Range (ug/mL)	Ampicillin MIC Range (µg/mL)
BW25113 (Parent Strain)		0.016	2-3
efp	Elongation factor (EF-P)	0.008-0.016	2-3
entA	2,3-dihydro-2,3-dihydroxybenzoate dehydrogenase	0.016	2-4
entS (*ybdA*)	Enterobactin efflux transporter EntS	0.008-0.016	2-4
epmA (*genX, poxA, yjeA*)	EF-P-lysine lysyltransferase	0.016-	2-5
fepA (*cbr, cbt, fep, feuB*)	Ferric enterobactin, colicin B, colicin D outer membrane porin	0.016	2-3
fetB (*ybbM*)	ABC transporter with a role in iron homeostasis membrane subunit	0.016	2-4
galK (*galA*)	Galactokinase	0.008-0.016	2-5
phoQ	PhoQ sensory histidine kinase	0.008-0.016	2
rffH (*yifG*)	dTDP-glucose pyrophosphorylase	0.016	2
soxS	SoxS DNA-binding transcriptional dual regulator	0.008-0.016	2-3
ugd (*yefA, udg, pmrE, pagA*)	UDP-glucose 6-dehydrogenase	0.016	2-4
waaB (*rfaB, lps, syn*)	UDP-D-galactose:(glucosyl)lipopolysaccharide-1,6-D-galactosyltransferase	0.016	2-4
wecA (*rfe*)	Undecaprenyl-phosphate α-N-acetylglucosaminyl transferase	0.08-0.016	2-3
wzzB (*rol, cld*)	regulator of length of O-antigen component of lipopolysaccharide chains	0.016	2-5
wzzE (*metNSt, yifC*)	Enterobacterial common antigen polysaccharide chain length modulation protein	0.016	2-4

[1] Gene Synonyms from EcoliWiki (http://ecoliwiki.net/colipedia/index.php?title=Category:Gene_List:MG1655&pageuntil=aspS%3AGene), and EcoCyc (https://ecocyc.org/)
